# Responding to whom? An experimental study of the dynamics of responsiveness to interest groups and the public

**DOI:** 10.1080/13501763.2024.2306226

**Published:** 2024-02-07

**Authors:** Anne Rasmussen, Simon Otjes

**Affiliations:** a Department of Political Economy, King's College London, London, UK; b Department of Political Science, University of Copenhagen, Copenhagen, Denmark; c Department of Comparative Politics, Bergen University, Bergen, Norway; d Institute of Political Science, Universiteit Leiden, Leiden, Netherlands

**Keywords:** Responsiveness, public opinion, interest groups, experiment, business groups, environmental groups

## Abstract

While politicians are commonly depicted as having strong incentives to be responsive to both interest groups and citizens to govern and maintain office, the literature lacks designs that allow for assessing the causal effect of both types of actors on individual policy-makers. This study addresses this gap by formulating theoretical propositions regarding responsiveness of politicians to both public opinion and interest groups and testing them in a vignette experiment with responses from over 2000 Danish and Dutch local, regional and national elected representatives. Our study finds important differences in the dynamics of responsiveness to the two types of actors: Public opinion has a strong direct effect on the intended voting behaviour of politicians, whereas the effects of interest groups are weaker and mainly demonstrate the potential to influence the views of ideologically aligned legislators. Left-wing politicians, in particular, are responsive to civil society groups. These results have implications for understanding political representation and the role of interest groups across multiple levels of government. While the heightened sensitivity of politicians to some aligned groups creates a risk of policy-making biases, it is reassuring that interest groups have a weaker effect than public opinion and primarily hold potential to influence like-minded politicians.

## Introduction

The job of elected representatives involves balancing a series of goals, such as working for their own re-election, ensuring that public policy reflects the public interest and securing the necessary technical and scientific input to adopt efficient decisions (Fenno, 2003). To effectively achieve these aims, elected representatives frequently rely on interactions with citizens and interest groups, who provide them with valuable exchange goods that help them both survive and continue their time in office in return for addressing the concerns of these stakeholders. Politicians are therefore not only incentivised to follow their own conscience, akin to what has been described as *trusteeship* (Burke, 1774), but also to be responsive to the opinions of both interest groups and the public.

Not surprisingly, studies have therefore increasingly incorporated both interest groups and public opinion in studies of agenda and policy responsiveness (e.g., Agnone, 2007; Bevan & Rasmussen, 2020; Burstein, 2014; De Bruycker & Rasmussen, 2021; Gilens, 2012; Klüver & Pickup, 2019; Lax & Phillips, 2012; Rasmussen *et al*., 2021). However, the dominant approach is to examine whether the public and interest groups attain their policy positions in the final policy outcomes. Even with an army of control variables, these observational analyses ultimately cannot show that policy-making is *driven* by interest groups or public opinion, since the same patterns could occur for a number of other reasons (Butler & Nickerson, 2011; Sevenans, 2021). Citizens could for example elect representatives that are aligned with them to begin with, or public opinion could be influenced by elected representatives rather than the other way around. Similarly, what might look like interest group influence could in reality be interest groups having adjusted their demands to what they deem feasible to obtain from policy-makers.

To tackle these challenges, studies of responsiveness to the public have started using experimental methods to determine the causal effects of obtaining information about public opinion on the attitudes and behaviour of elected representatives. This includes research looking at the responsiveness of political elites to constituent communication (Costa, 2017) as well as research on how politicians respond to different public opinion signals. Butler and Nickerson (2011) show that providing state representatives with information about their constituents’ policy preferences increases their likelihood of casting a vote in line with public opinion. Sevenans (2021) demonstrates that a potential mechanism driving such responsiveness in behaviour might be a capacity for the public to first affect the opinion of legislators.

Despite this ability to offer important resources to decision-makers, the empirical study of interest groups has often found no evidence for interest group influence (Leech, 2010; Lowery, 2013). This could in part be the result of how scholars have approached the study of interest group influence: the experimental study of lobbying may help isolate factors associated with influence. Such research however is relatively rare (Lowery, 2013). Recent studies include survey experiments, assessing the responsiveness of public opinion to arguments by different types of interest groups (Dür, 2019), legislators’ receptiveness to grassroots lobbying (Cluverius, 2017), and the impact of individual citizen and interest group contacts on legislative staffers’ responsiveness and perceptions of public opinion (Furnas *et al*., 2023; Hertel-Fernandez *et al*., 2019). Other studies use field experiments, with groups attempting to obtain access to political offices through phone calls (Brodbeck *et al*., 2013) and campaign contributions (Kalla & Broockman, 2016), or influence legislators’ policy positions or voting behaviour through different forms of direct lobbying (Grose *et al*., 2022) and via email (Bergan, 2009). Additional field experiments analyse responses to lobbying letters by citizen groups (Richardson & John, 2012), and test the ability of interest groups to persuade citizens (Jungherr *et al.*, 2021; Junk & Rasmussen, 2023).

The field is still in need of comprehensive experimental designs that explore policy responsiveness of individual politicians to *both* interest groups and public opinion. We present such a design to study whether learning about both public opinion *and* different interest group preferences on a specific policy issue affects the intended voting behaviour of politicians. By including both public opinion and interest groups simultaneously, we are able to study, not only whether these two factors matter but also, which is more important. Moreover, it allows us to empirically investigate variation in the nature of policy responsiveness to public opinion and interest groups. Finally, by varying the policy positions of both the public and different interest groups in a credible way, we can examine, not only whether the opinions of the public and interest groups matter, but also whether their impact depends on their positions.

We argue that elected representatives have incentives to be responsive to both citizens and interest groups, which both provide representatives with important goods that help them fulfil their mandate and maintain office. Yet, we employ theories of social identification and motivated reasoning to argue that, in the case of interest groups, responsiveness is likely to be selective and ideologically conditioned.

We test our predictions in a vignette experiment with responses from over 2000 politicians at the local, regional and national level in Denmark and the Netherlands. We show that politicians are highly sensitive to public support for a given policy proposal and that interest groups can also affect politicians’ votes. However, the direct impact of groups is weaker and not consistently significant across samples and model specifications. Instead, we find support for our theory that responsiveness to interest groups is more likely to be selective and ideologically conditioned: Left-wing politicians in particular adapt their behaviour to positions of civil society groups (specifically environmental groups). In contrast, the influence of business groups on right-wing politicians appears to be less pronounced.

Our findings have important implications for enhancing our understanding of the role of interest groups in policy representation. They illustrate the risk that politicians are not only affected by the views of the public but also by interest groups who might champion causes different from those of average citizens. Yet, by demonstrating that interest groups predominantly have potential to sway like-minded representatives, and that their overall influence on politicians is weaker than that of public opinion, these findings also serve to alleviate potential concerns about their undue influence.

## Theory and hypotheses

Seen from a resource exchange perspective (Bouwen, 2004; Pfeffer & Salancik, 1978), the reason decision-makers should not only follow their own conscience (Burke, 1774) but also pay attention to the electorate and interest groups is that these two types of actors have something to offer decision-makers in exchange for possible influence.

### Public opinion

The public might possess the most crucial resource that representatives desire: the vote that can help politicians and/or governments get (re-)elected (Mayhew, 1974). The re-election incentive is therefore the dominant explanation for why politicians should be responsive to the views of the public in policy-making (e.g. Canes-Wrone *et al*., 2002; Manza & Cook, 2002). Burstein (2014, p. 107) argues that this gives the public ‘the ultimate authority over policy’. Yet, research does not always find a perfect match between what the public desires and the state of policy. In one of the most comprehensive studies of opinion-policy congruence in the US (aptly titled the ‘Democratic Deficit in the States’), Lax and Phillips (2012) show that state policy is congruent with what the majority of the public wants only approximately half of the time. In a similar study on Europe (Rasmussen *et al*., 2019), the score is somewhat higher, i.e., 63 per cent, but also far from indicating that legislators simply do what the public wants. Yet, these studies do find that higher public support for a given policy increases the likelihood that it is in place, albeit the relationships are not always strong. Furthermore, both observational and experimental research provide evidence that, not only collective policy outputs, but also individual decisions of policy-makers are to some degree related to public opinion (Butler & Nickerson, 2011; Hanretty *et al*., 2017). Apart from maximising chances of re-election, elected representatives may also be incentivised to be responsive to public opinion based on a sense of duty or moral responsibility to serve the public (Fenno, 2003).^1^ This leads to our first hypothesis expecting a positive relationship between the degree of public support for a proposal and the intention of policy-makers to support it:

1. *Public opinion hypothesis:* The larger the public opinion majority that supports or dislikes a policy proposal, the more likely politicians are to take positions that are congruent with public opinion.

### Interest groups

The re-election incentive should put interest groups at a disadvantage in seeking political influence as they typically only represent a segment of the public (Flöthe & Rasmussen, 2019). However, the general public does not care equally about all policy issues and politicians do not need every single vote to get re-elected. Certain interest groups may represent specific subsets of the public, critical to elected representatives from particular parties for both re-election and successful implementation of adopted policies. Some interest groups can even be sources of broad public appeal in the policy areas they represent. Interest groups also offer a number of other resources valuable to policy-makers, potentially influencing elected representatives to align with their policy stances (Berkhout, 2013). These include technical and legal expertise crucial for politicians to adopt specific policies (Bouwen, 2004; Burstein, 2014; De Bruycker, 2015; Eising, 2007; Mahoney & Beckstrand, 2011). Their advice may not be ‘neutral’ but may help decision makers adopt complex policy decisions when their time and their existing knowledge of issues are limited.

Even if any given interest group type may hold a combination of the resources mentioned, the relative possession of and dependence on these types of resources is often expected to vary between different group types (e.g., Eady & Rasmussen, 2022; Rasmussen & Reher, 2023). Civil society groups representing broader societal interests are key providers of (broad) public appeal and legitimacy (Mahoney & Beckstrand, 2011). Conversely, business associations are often portrayed as having a comparative advantage when it comes to offering specialised, technical information (Bouwen, 2004). Politicians are typically interested both in boosting public legitimacy and in obtaining technical input for making informed decisions on specific policies. This means that, even if the nature of the resource exchange of elected representatives with civil society and business groups may vary, the resources offered by these two types of groups are typically complementary. As a result, we would expect politicians to be responsive to the positions expressed by both civil society and business groups:

2. *Civil society interest hypothesis:* Politicians are more likely to support policy proposals that are supported by interest groups representing civil society interests than proposals that are not.

3. *Business interest hypothesis:* Politicians are more likely to support policy proposals that are supported by interest groups representing business interests than proposals that are not.

Rather than having a general ability to affect policy-makers, we can also imagine the impact of these different interest groups to be dependent on the ideological connections between politicians and different interest groups, such as civil society groups and business groups. Interest groups themselves take positions in the political space that may be closer to some parties than others (Boräng *et al*., 2023). A natural affinity exists between business groups and the ideological right, and between many civil society organisations and the ideological left (Aizenberg, 2023; Allern *et al*., 2022; Berkhout *et al*., 2019; De Bruycker & Rasmussen, 2021; Otjes & Rasmussen, 2017). Both business groups and the ideological right believe that the free market is the best way to allocate value: when companies profit, they can employ workers. Therefore, they are both opposed to excessive market regulation. Several civil society organisations and the ideological left, on the other hand, both emphasise that there are other values than the maximisation of profit for business, such as in our case, the intrinsic value of nature.

Politicians are likely to respond more strongly to information from the types of interest groups, with which they are ideologically aligned for three reasons. Firstly, politicians are likely to feel a stronger sense of identification with these groups and therefore take positional cues from them more seriously. In the terminology of social identification theory (Tajfel & Turner, 1986), some elected representatives and ideologically aligned interest groups can be considered part of the same in-group. This provides elected representatives with an incentive to respond to such interest groups at the expense of other groups, which could be considered as more of an out-group for them.

Secondly, politicians are not only more likely to hold an overall ideological connection to certain types of groups but are also more likely to agree with these groups on specific issues. In line with the political-psychological theory of motivated reasoning (Kunda, 1990), politicians may be driven by directional goals and filter information from specific sources differently in order to arrive at opinions in line with existing values and beliefs. For instance, politicians discount the opinion of citizens with which they disagree and reasoning that these citizens are less informed (Butler & Dynes, 2016). A similar dynamic might be at play when interpreting evidence from interest groups. US congressional staff for example has a higher propensity to side with and use evidence from think tanks with which these staffers are ideologically aligned (Furnas *et al*., 2023).

Thirdly, elected representatives may respond more strongly to information from ideologically aligned interest groups to maximise their chances of re-election. Electoral incentives affect responsiveness (Soontjens & Sevenans, 2022), and politicians may anticipate a higher chance of being held electorally accountable when reacting to information from ideologically aligned interest groups. Idelogically aligned interest groups are more likely to both represent subsets of the public that vote for the party and hold information about the policy positions of these constituencies. These arguments lead us to formulate our final hypotheses:

4. *Business interest-right hypothesis:* Business groups have a greater impact on the positions of right-wing than left-wing politicians.

5. *Civil society interest-left hypothesis:* Civil society organizations have a greater impact on the positions of left-wing than right-wing politicians.

## Methods

We test these expectations using a vignette survey experiment conducted among all elected representatives at the local, regional and national level in Denmark and the Netherlands.^2^

### Case selection

Our choice to use a two-country design is primarily motivated by a desire to ensure that our findings are robust to the idiosyncrasies of specific political systems rather than an attempt to explain cross-national variation in responsiveness. At the same time, the Netherlands and Denmark share commonalities in the links between elected representatives and citizens making them sufficiently similar for a pooled analysis. Both countries are unitary states and have proportional electoral systems, leading to diverse multi-party systems. In both countries there are structured, neo-corporatist relationships between key interest groups and the government (Jahn, 2016). Both also have a uniform three-tiered government system with municipalities, regions and national government.^3^ All three levels play a role in decision-making regarding the topic of our experiment: land use and zoning policy. Finally, both countries also have a parliamentary system at every level of government: the national/municipal/regional council is elected by the voters and in turn elects an executive at their level.

### Respondents

Our sample of representatives includes all members of the Danish *Folketing* and the Dutch *Tweede Kamer der Staten-Generaal* as well as the Danish and Dutch regional councils and the municipal councils. Altogether, we have a sample of 11,889 politicians from which we received 2994 responses, corresponding to a 25 per cent response rate.^4^ Table A1 in Appendix shows the response rates for the different subsamples. Table A2 shows that all party families and men and women are represented in roughly the same proportions as the population of politicians. The survey experiment ran online from 18 March 2020 to 3 August 2020, and was part of a larger project on representation.^5^

### Experiment

Our experiment focuses on the placement of windmills on land with treatments for both public opinion and the positions of civil society and business groups on the issue. Both in the Netherlands and Denmark, this is a realistic case, as a large number of windmills on land are being considered, and the precise placement of windmills is subject to considerable politicisation (Devlin, 2005; Ladenburg, 2015; Wolsink, 2010). We use environmental groups as an example of civil society interests due to their emphasis on defending broader societal interests of a more diffuse character in contrast to the economic interests defended by business groups (Baroni *et al*., 2014).

Each politician was asked to imagine a scenario with a proposal regarding the construction of a new windfarm on land (see Box 1). This windfarm would be located in their municipality, region or country depending on the level of government of the politician in question. The vignette exposed politicians to a random combination of three treatments about the support for the policy among (1) voters, (2) civil society groups and (3) business groups. For voters, we provided information about public support for the proposal with different levels of support ranging from 35 to 65 per cent (leading to five options, including absence of information on public opinion).^6^ For civil society and business groups, we provided information about their opinion towards the proposal (either support or opposition). Because industry and environmental groups can favour and oppose wind energy, this allows us to manipulate the interest groups’ positions in the experiment in a realistic way to determine whether the impact of groups depends on the opposition or support of a given policy.^7^ Some environmental groups might favour windfarms as a carbon-neutral energy source but others may oppose windfarms because of their effect on the surrounding nature, including the visual impact on the landscape and bird and bat casualties (Brandt & Svendsen, 2004; Nichifor, 2016). Similarly, business groups that own, operate or sell windmills will support them while those representing coal or nuclear industry groups might oppose them, as may tourism-dependent businesses. Apart from being in favour or against, we also vary how large a share of the relevant stakeholders these interest groups represent, which we analyse in the Appendix. With three options for the latter, this leads to seven options per group type, including a scenario where a given type of group is not mentioned in the vignette. Our fully randomised 5 × 7x7 factorial design therefore ends up with 245 conditions.

Box 1. **Vignette.**

Below we will give a description of a fictitious policy case.

Imagine that you are your party’s spokesperson on spatial planning issues and need to make a decision on the case. Please read the description carefully before answering a few questions about your view of the decision. To make sure you have enough time to consider your answers, it will be 15 s before you see the arrow that lets you get to the next page. Of course, you can spend as much time answering the questions as you need.

Your [municipal council/regional council/national parliament] is considering whether to allow for a new windfarm on land [in your municipality/in your region/in the Netherlands/Denmark].

You have read a report about a reliable opinion poll that suggests that {35/45/55/65} per cent of the voters in your municipality support the proposal {leave out sentence}.

Environmental groups representing {5/10/25} per cent of the citizens in your municipality/region/country have expressed {support for/opposition to} the proposal {leave out sentence}.

Business groups representing {5/10/25} per cent of the businesses in your municipality/region/country have expressed {support for/opposition to} the proposal {leave out sentence}.

Text in curly brackets changed between vignettes and text in brackets changed between samples.

The assignment of the vignettes was randomised within Qualtrics. Appendix A3 displays balance tests for self-identified gender, age, left-right self-placement, the prior agreement measure, education, country and level of government. They do not lead us to believe that the results were biased by the assignment.

In an attempt to ensure that respondents read the vignette, the survey was halted for 15 seconds when respondents reached the vignette. We also included manipulation checks asking respondents about the subject of the vignette they read, as well as the position of environmental groups, business groups and the voters in this vignette. Our experiment was pre-registered with EGAP before we accessed the data.^8^

A survey experiment such as ours is an abstraction of a rich political reality. Compared to an observational design, we can attribute effects on intended voting behaviour to our treatments without having to account for the background characteristics and the complex endogenous feedback mechanisms that might play a role in representation. We choose for a survey experiment over a field experiment. Firstly, a field experiment with interest groups contacting a large number of elected representatives within the same elected body aiming at influencing their opinions and/or behaviour on a specific policy issue likely has relatively low external validity in the political systems examined. Due to a high degree of specialisation and party discipline among representatives, direct lobbying is typically not done through mass appeals but by targeting relatively few legislators that act as spokespersons on behalf of their political party for a given policy. In qualitative interviews we conducted for the wider project, a lobbyist even mentioned how (the few) mass appeals experienced were counter-productive for exerting influence as they were interpreted as examples of desperate lobbying without sufficient knowledge of the state-of-play. Secondly, obtaining actual public opinion and interest group position data not only for a given country but also for all of its municipalities and regions would not have been practically feasible. Moreover, we are not only interested in receiving information about whether (a specific level of) public support matters but also about how our outcome variable is assessed for different levels of public support. Similarly, we do not just want to know whether the two types of interest groups in a given district can affect intended voting behaviour but also whether these groups are more or less influential when they are in favour or against. In our design, we can vary the information that different representatives within the same local, regional or national district receive about public opinion and interest group positions.

### Method of analysis

Our dependent variable is the likelihood that a representative will vote for the windfarm proposal. ‘Could you indicate on the slider below how likely you would be to vote in favour of the proposal described?’ on a scale from 0 to 100. We choose ‘intended voting behaviour’ of the respondents rather than ask them about their opinion towards a policy issue after the intervention, as policy responsiveness involves something beyond an attitudinal response. We acknowledge that politicians in proportional systems are frequently constrained by their party in their final vote. Rather than looking at the actual vote choice, our intended measure instead taps into what elected representatives would prefer to vote prior to coordination (Sevenans, 2021).

To see whether ideological alignment of the MPs affects the impact of different types of interest groups, we compared the effects of learning about the support of environmental and business groups for politicians at different points along the left-right dimension.^9^ The descriptives of the variables are included in Appendix A2.

The analyses in the main paper were run as Ordinary Least-Squares Regressions, which are frequently used to analyse full factorial experimental designs (Furnas *et al*., 2023; Hainmueller *et al*., 2014; Wallander, 2009). We include dummies for each of the country-government level combinations to pick up on differences in the prior agreement among the samples. We present models with and without the respondents’ prior attitude towards windmills, which we asked earlier in the survey.^10^

As discussed above, we checked whether respondents were able to reproduce the manipulation. In the paper, we show the analyses for both this subsample and the full sample of all the treated respondents. The results for two groups of respondents generally show the same pattern, with the effects for those who answered the manipulation checks correctly being stronger and more often highly significant. 2994 respondents participated in the survey, and 2067 of those completed the questionnaire at least until the experiment. This is 17 per cent of all invited respondents and 69 per cent of those who started the survey. 1290 of those 2067 respondents were able to complete *all four* manipulation checks correctly.^11^

## Results

**Figure 1.**
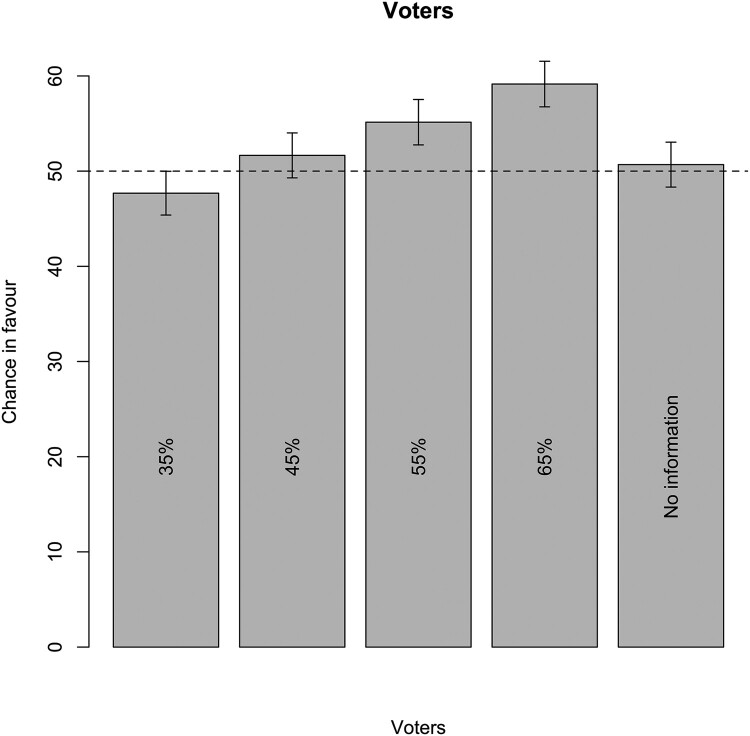
Chance in favour and positions of voters. Notes: Based on Model 6; expected values and 95 per cent confidence intervals. Line at 50 per cent. Note that scale differs from Figures 2 and 3.

**Figure 2.**
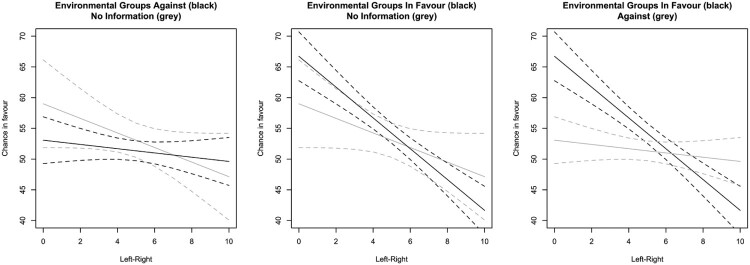
Chance in favour and positions of environmental groups and left-right positions. Notes: Based on Model 6; expected values and 95 per cent confidence intervals. Note that scale differs from Figures 1 and 2.

**Figure 3.**
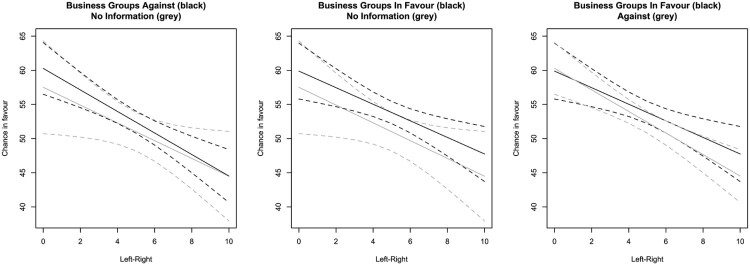
Chance in favour and positions of business groups and left-right positions. Notes: Based on Model 6; expected values and 95 per cent confidence intervals. Note that scale differs from Figures 2 and 3.

Table 1 presents eight regressions with the same outcome variable. The first models examine only the main effect of interest groups, whereas the last four add the interaction between the left-right dimension and the two types of interest group positions. For each set of models, we present results for *all respondents* and for only those *respondents that passed all manipulation checks*. We also run the analyses with and without a measure of prior agreement controlling for the politician’s own position towards the issue. Figures 1–3 visualise the regression results for all respondents and including prior agreement.

**Table 1.** Regression models.

Model	1	2	3	4	5	6	7	8
Sample	All	All	MC	MC	All	All	MC	MC
Intercept	67.66***	10.78***	69.65***	12.88***	63.54***	6.51*	66.58***	9.49**
	(2.62)	(2.78)	(3.13)	(3.46)	(3.46)	(3.33)	(4.09)	(4.06)
Prior agreement		13.77***		13.38***		13.76***		13.34***
		(0.43)		(0.55)		(0.43)		(0.54)
Left-Right	–3.99***	–1.39***	–4.35***	–1.68***	–3.18***	–0.54	–3.86***	–1.07**
	(0.29)	(0.25)	(0.34)	(0.30)	(0.54)	(0.45)	(0.64)	(0.54)
Voters^a^ =								
45%	4.20**	3.68**	3.31	3.92*	4.52**	3.97**	3.88	4.38**
	(2.08)	(1.69)	(2.44)	(2.00)	(2.08)	(1.68)	(2.43)	(1.99)
55%	7.30***	7.12***	8.41***	8.98***	7.66***	7.46***	8.91***	9.46***
	(2.08)	(1.69)	(2.50)	(2.06)	(2.08)	(1.69)	(2.49)	(2.04)
65%	10.52***	11.38***	10.51***	10.66***	10.59***	11.46***	10.92***	11.01***
	(2.10)	(1.70)	(2.47)	(2.03)	(2.09)	(1.69)	(2.46)	(2.01)
No information	3.77*	3.04*	1.57	2.50	3.71*	3.00*	1.47	2.40
	(2.08)	(1.69)	(2.77)	(2.28)	(2.08)	(1.68)	(2.76)	(2.26)
Environmental Groups^b^ =								
In favour	1.90	2.84**	2.29	3.60**	13.18***	13.65***	15.25***	16.77***
	(1.44)	(1.17)	(1.75)	(1.44)	(3.39)	(2.75)	(4.00)	(3.26)
No information	2.83	1.82	0.48	1.58	6.60	5.92	15.62**	13.20**
	(2.02)	(1.64)	(2.63)	(2.16)	(5.04)	(4.07)	(6.27)	(5.13)
In favour * Left-Right					–2.25***	–2.16***	–2.57***	–2.61***
					(0.62)	(0.50)	(0.73)	(0.59)
No information * Left-Right					–0.77	–0.84	–3.15***	–2.42**
					(0.92)	(0.75)	(1.20)	(0.98)
Business Groups^c^ =								
In favour	1.71	1.62	4.49**	3.72**	–0.65	–0.39	–5.47	–4.97
	(1.44)	(1.17)	(1.79)	(1.47)	(3.42)	(2.78)	(4.13)	(3.37)
No information	–1.04	–1.53	2.41	1.18	–3.76	–2.76	–2.94	–1.73
	(2.05)	(1.66)	(2.39)	(1.96)	(4.86)	(3.93)	(5.45)	(4.44)
In favour * Left-Right					0.43	0.36	1.95**	1.68***
					(0.62)	(0.51)	(0.76)	(0.62)
No information * Left-Right					0.57	0.27	1.09	0.61
					(0.87)	(0.70)	(0.99)	(0.81)
Sample^d^ =								
Danish National	7.58	6.17	6.07	4.75	7.40	6.02	6.10	4.78
	(7.41)	(6.00)	(8.42)	(6.89)	(7.39)	(5.97)	(8.36)	(6.82)
Danish Regional	2.39	0.09	2.77	0.81	2.62	0.33	3.79	1.79
	(4.06)	(3.31)	(5.35)	(4.38)	(4.06)	(3.31)	(5.32)	(4.34)
Dutch Municipal	–2.30	–4.62***	–3.12	–5.06***	–2.21	–4.52***	–2.68	–4.68***
	(1.66)	(1.35)	(2.03)	(1.67)	(1.65)	(1.34)	(2.01)	(1.65)
Dutch National	5.81	0.52	9.76	5.25	4.47	–0.99	8.97	3.84
	(9.46)	(7.66)	(10.42)	(8.53)	(9.50)	(7.68)	(10.38)	(8.47)
Dutch Regional	–6.95**	–1.95	–8.72**	–3.67	–6.83**	–1.84	–8.28**	–3.25
	(2.98)	(2.42)	(3.52)	(2.89)	(2.97)	(2.41)	(3.49)	(2.86)
*R*-squared	0.11	0.42	0.15	0.43	0.12	0.43	0.16	0.44
*N*	1921	1904	1230	1222	1921	1904	1230	1222

Notes: Ordinary Least Squares Regression. All = All respondents and MC = Respondents who answered all the manipulation checks correctly. ^a^Reference category: voters = 35 per cent; ^b^Reference category: Environmental Groups = Against ^c^Reference category: Business Groups = Against; ^d^Reference category: Danish Municipal; 0.1 > * > 0.05 > ** > 0.01 > ***.

The public opinion hypothesis proposed that the larger the public opinion majority that supports or dislikes a policy proposal, the more likely politicians are to take positions that are congruent with public opinion. Figure 1 shows the average chance of voting in favour of the proposal for different conditions without controls. Firstly, we compare the first and second scenario. If, as in the second scenario 45 per cent of the citizens favours the placement of windmills, the majority of respondents (52 per cent) favours their placement. If popular support decreases to 35 per cent, the support among politicians decreases (to 48 per cent in favour). This decrease in intention to support the windfarm among the politicians between the second and the first scenarios is significant and in line with the Public opinion hypothesis. The third and the fourth scenarios show an increase in public support for the placing of windmills from 55 to 65 per cent. This is associated with an increasing chance that politicians favour placement from 55 to 59 per cent. This increase is also significant and in line with the Public opinion hypothesis. Both comparisons support the notion that when a larger share of the public favours a measure, the likelihood that politicians take a congruent position increases.

Moreover, there is also strong evidence demonstrating elected representatives’ responsiveness to public opinion overall. The 55 per cent chance that politicians favour placement with 55 per cent of voters favouring it in the third scenario represents a significant increase of three percentage points compared to the second scenario (i.e., with 45 per cent of the voters supporting the measure). In general, these results provide evidence that there is a relationship between public support for a proposal and the extent to which politicians intend to vote in favour.^12^

In contrast, the hypotheses that the positions of interest groups representing business and civil societal interests affect the intended voting behaviour of the politicians do not receive consistent support in Table 1. We do see small but significant effects under specific conditions: in particular when business groups favour the measure, the chance of support increases by between one and five percentage points compared to when they are against (Models 3 and 4). However, this effect is only significant among the sample of respondents who passed the manipulation check, in which we cannot be sure that the treatments are truly randomly assigned. For environmental groups, the effect is of a similar size (between one and four percentage points) but it is only significant when controlling for prior agreement (Models 2 and 4). The situation where there is no information does not differ significantly from *any* of the other experimental conditions in the different models for both environmental and business groups (at the 0.05-level).

Finally, we test the Business interest-right hypothesis and Civil society interest-left hypothesis proposing that left-wing politicians are more likely to take the concerns of environmental groups into consideration, while right-wing politicians are more receptive to the concerns of business groups. Table 1 includes these regressions in Models 5–8 and Figures 2 and 3 visualise the patterns based on Model 6. For environmental groups, these results are substantial and significant. When respondents are informed that environmental groups favour windmills, politicians with the most left-wing position have a 67 per cent chance to favour it compared to 42 per cent of the politicians with the most right-wing position. When politicians are informed that environmental groups oppose the placement of windmills, we also see a stark shift in opinion: the support among left-wing politicians decreases to 53 per cent (a decrease of 14 percentage points) and the support among right-wing politicians increases to 50 per cent (an increase of 8 percentage points). Under this circumstance, the right and the left are statistically equally likely to support the measure. This result supports the notion that the cues of environmental groups affect the support of left-wing politicians for windmills without a similar effect for right-wing politicians.^13^

Without information about the position of environmental groups, left-wing politicians have a 59 per cent chance to favour placing windmills compared to a 47 per cent chance for right-wing politicians, but note that these results for the respondents who did not get cues from interest groups show greater levels of uncertainty.

For business groups, we do not find a significant pattern in Figure 3. If respondents are informed that business groups oppose the measure, the support among left-wing politicians is 60 per cent. Among the right, it is 45 per cent. If politicians are informed that business groups favour the measure, support among left-wing groups remains 60 per cent. Among right-wing politicians, the support increases to 48 per cent in line with our theoretical expectations. Yet, this increase is not significant. In the analysis excluding those who failed the manipulation checks, there is a significant effect. Finally, if no information on business groups is provided, the support among left-wing politicians is 58 per cent, and among right-wing politicians it is 44 per cent.

### Robustness tests

In Appendix A5, we test the robustness of the results by running a number of additional models. The first ones look at different set-ups for dealing with the multilevel structure of the data. Table A7 presents a multi-level model with different country*government level combinations as a second level in order to deal with unobserved structural differences between the populations. These results are similar to those presented in the main paper. Table A8 only analyses the Dutch data. These results support those of the main paper. Table A9 presents the Danish results. These estimates come with greater uncertainty than the Dutch, given that the Dutch sample is three times larger than the Danish one. As can be seen in Figures A1–A7, for all three types of representatives we find slightly different results: firstly, the effect of public opinion is not as linear as it is in the Netherlands (that is the effect of public opinion being at 55 per cent or 65 per cent is statistically indistinguishable). Secondly, environmental groups have stronger direct effects on support. When they are in favour, centre and centre-right politicians favour windmills more than Dutch politicians. Table A10 presents the data for the municipal councillors in both countries. These conform to those presented in the paper. Table A11 presents the data for the regional and the national politicians. The sample size of this subset is an order of magnitude smaller than the size of the sample as a whole; therefore, it is associated with higher levels of uncertainty. Figures A8–A14, show that the only pattern that remains significant in this smaller sample is the conditional effect of environmental groups on left-wing politicians (and even then, only in some models and with considerably lower levels of significance). All in all, the effect of public opinion is weaker among non-municipal politicians, compared to municipal politicians.^14^

Table A12 presents the effect of public opinion separately. These effects are the same as in the paper. Tables A13 and A14 do the same for environmental and business groups, again replicating the results of the multivariate models in the paper. Tables A15 and A16 present exploratory tests for the possibility of an interaction between public opinion and either the politicians’ own policy preference, their left-right ideological position, or the extremity of their ideological position, but these results are not significant. Responsiveness to general public opinion does not systematically vary for representatives with different positions towards windfarms, different positions on the left-right dimension, or between those holding extreme and centrist ideological positions. Table A17 dichotomises the public opinion variable. This allows for a direct comparison of public opinion and group effects: the effect of public opinion moving from being opposed to being favour of the windfarms on politicians’ intention to support them is between seven and nine points, while for environmental groups it is between zero and four points and for business groups between one and five points. In the specifications where the direct effects for groups are significant, the effect of public opinion is thus at least double as high as that of interest groups. In Table A18, we explore potential interactions between public opinion and interest groups on elected representatives. Figure A17 shows that, regardless of interest groups’ stance, public support consistently influences politicians’ backing for windmills, indicating that the impact of public opinion and interest groups is not contingent on each other.

### Other pre-registered tests

As mentioned, our pre-registration included three additional hypotheses regarding other potential heterogeneous treatment effects for groups, which we discuss in Appendix A6. These concern expectations that politicians are more responsive to groups, the more representative these groups are of their potential stakeholders (Hypothesis A1), and the stronger their level of engagement in business (Hypothesis A2) or civil society groups (Hypothesis A3). The tests of A1 show that the representativeness of the group does not consistently moderate the effect of group position on the voting behaviour of politicians. Similarly, the tests of A2 show that business group ties do not moderate the effect of business group position on voting behaviour. Finally, the tests of A3 are more promising (politicians with links to environmental groups are more sensitive to their position than those without) but this effect disappears once we include the interaction between these groups and the ideology of politicians (cf. Civil society interest-left hypothesis). These findings indicate that left-right positions and engagement with environmental groups are not independent of each other. They also highlight that the primary factor influencing the conditioning effect of environmental groups on the responsiveness of politicians is their ideology.

## Conclusion

The inquiry into which forces affect elected representatives when making policy decisions has been a key concern to political scientists since the early days of the discipline. A voluminous literature has examined the relationship between public opinion and either the individual or collective decisions of elected representatives (e.g. Lax & Phillips, 2012; Miller & Stokes, 1963; Rasmussen *et al*., 2019). In recent years a new literature has innovative, experimental designs to the previous efforts. They have aimed at identifying the causal effects of learning about public preferences on the behaviour of politicians, often with a focus on responses to requests for *service* as opposed to actual *policy* responsiveness (Costa, 2017; but see Butler & Nickerson, 2011; Soontjens & Sevenans, 2022). In the interest group literature, causal designs have also gained prominence, even if many efforts have been directed at examining the causal impact of groups on *citizens* rather than on *politicians* (Dür, 2019; Jungherr *et al*., 2021; Junk & Rasmussen, 2023).

We present a comprehensive experimental design that taps into the policy responsiveness of individual representatives *both* to public opinion and interest groups. While we expect all elected representatives to have incentives to respond to public opinion, we use theories of social identification and motivated reasoning to argue that politicians should primarily be selective in their responses to interest groups, prioritising groups with which they are ideologically aligned.

In line with our expectations, we find a strong general tendency for politicians to be sensitive to public support when stating their intended voting behaviour on a given policy issue. For interest groups the story is somewhat different: learning about interest group positions also affects the intended voting behaviour of elected representatives, but these effects are not consistently found in the different samples and model specifications. Instead, there is more support for our theory that responsiveness to interest groups is selective and ideologically conditioned. The potential of groups to persuade politicians how to vote is for the most part restricted to ideologically aligned interest groups. This conditional effect suggests that identification, shared policy opinions and joint constituencies with interest groups matter for politicians when deciding whether to be receptive to the opinions of interest groups in practice. Especially civil society groups show a capacity to primarily trigger responses among the most left-wing politicians, while the evidence that business groups to elicit responses from right-wing politicians is much weaker. This might be related to the fact that a politician’s relationship to business groups is less ‘emotional’ in nature than to civil society groups. The latter are typically regarded as working not only for the provision of material benefits (Olson, 1965) but also to promote solidary and purposive goods, e.g., a feeling of social status, a sense of companionship or promotion of broader supra-personal goals (Moe, 1981; Salisbury, 1969). It is possible that this might create a stronger sense of identification between left-wing politicians and civil society groups than we see among right-wing politicians and business groups.

From a democratic point of view, our results present both good and bad news. It should be reassuring to learn that the direct influence of interest groups is weaker than that of public opinion. After all, most of these groups – even those representing societal interests – do not represent the view of the general public and some groups of privileged citizens are better represented by interests groups than the public as a whole (Boräng & Naurin, 2022; Hanegraaff *et al*., 2022). However, we observed that some interest groups have the capacity to influence politicians, especially those aligned ideologically, with the risk that these interest groups may sway like-minded politicians into adopting policies that diverge from the general public opinion.

These findings add considerably to the existing body of work on political representation while leaving scope for extensions in future research. Any survey experiment is an abstraction of empirical reality and should be seen as a complement to both observational research and the emerging body of field research that touches upon similar issues. Given our interest in ‘policy’ as opposed to ‘service’ responsiveness, we did not deem it feasible to conduct a field experiment in collaboration with an interest group in the political systems examined (Grose *et al*., 2022). Because of a high degree of specialisation and party discipline among representatives in Denmark and the Netherlands, professional interest groups generally use direct lobbying to target a smaller set of representatives that act as spokespersons on behalf of their party on an issue rather than issue mass appeals to a high number of representatives. At the same time, future field experiments – testing other forms of lobbying and employing alternative outcome measures – might be able to integrate both interest group and citizen treatments in a realistic way. Importantly, such field experiments on politicians would need to carefully consider important ethical issues in relation to consent, deception and debriefing (Zittel *et al*., 2023).

There is also room for additional survey experiments that dig closer into the mechanisms driving the different forms of responsiveness examined here. It may be possible to credibly present politicians with stimuli for the electoral importance of different types of interest groups or examine the impact of groups subject to the kind of information they provide. Such experiments could also collect information about representatives’ perceptions of public opinion versus interest groups to help determine whether social desirability plays a role in accounting for some of the differences in overall levels of responsiveness towards them. Research could also explore variation in responsiveness towards the opinion of the general public versus party constituents. Romeijn (2020) shows that when discrepancies between the two arise, parties tend to align with their party constituents. However, he also shows that such preference conflicts between general public opinion and party constituents only arise in about one in ten cases. Consequently, the trade-off of deciding which of the two to respond to is not a dilemma that politicians commonly encounter during the majority of policy-making processes. Nonetheless, the 10 per cent of issues where the median voter and the party constituents diverge are likely significant. Therefore, it is valuable to examine this potential trade-off further.^15^

Finally, while a strength of our design was to present a policy issue relevant to representatives at all levels of governance where we could credibly vary the positions of both interest groups and the public, there is also scope for extending our research to include different policy issues, other political systems, and public opinion signals for different subsets of citizens. What is clear, however, is that politicians do not only follow their own conscience but are responsive to both public opinion and interest groups, even if responsiveness to the latter is primarily ideologically conditioned.

## Supplementary Material

Supplemental Material

## Notes

1 In representative democracies, there are different options for defining the public, which could refer to both the electorate as a whole and the electorate of the representative’s party (Ezrow *et al*., 2011; Sevenans, 2021). In our public opinion treatments, we focus on the former, as it holds relevance even within proportional systems (Ezrow *et al*., 2011) and allows for direct comparisons with international literature. Our expectations regarding the selective nature of interest group responsiveness below also consider the party electorate by arguing that politicians listen more to ideologically aligned groups due to their ability to mobilise a party’s core electorate.

2 The preregistration with the Open Science Foundation can be found at https://osf.io/xmtuh/?view_only=ae37cb315e4049909db82edcdbf9dc2b.

3 The Netherlands has 12 regions (*provincies*) and, at the time of the survey, 355 municipalities (*gemeenten*). Denmark has five regions (*regioner*) and 98 municipalities (*kommuner*).

4 The Danish response rate is structurally higher than the Dutch response rate. The largest segment of respondents (about two-thirds) comes from Dutch municipal councils. Our response rate for the Dutch municipal level (23 per cent) is more than twice as large as the response rate of the National Municipal Councillors’ Study, a long-running survey, financed by the Dutch ministry of Home Affairs and Kingdom Relations and organised with the support of the Dutch association of municipal councillors. This 2021 survey had a 9 per cent response rate (Daadkracht, 2021). Our response rate is acceptable compared to other elite surveys (Bailer, 2014) even if its magnitude matters less for our experiment where validity comes from the randomisation of the stimuli.

5 Our wider project included a survey of citizens as well. Given that we wanted citizen and representative surveys in as close proximity as possible, and taking into account the initiation of citizen surveys in early March 2020, we made the decision to move forward with the representatives’ survey, even in the face of Covid-19 lockdowns. We cannot rule out that the pandemic has affected the response rates, even if we could both imagine decreases (as politicians needed to address health or family issues) and increases (as elected bodies had fewer issues on the agenda). Peyton *et al*. (2022) indicate that the pandemic does not pose a threat to generalisability of experiments with citizens, but may have resulted in smaller estimated treatment effects as subjects were less attentive. We might see a similar effect among elected representatives even if elite respondents might be less prone to experiencing such an effect. Importantly, our case issue (energy policy) is not directly related to the pandemic.

6 To assess citizen responsiveness, we opted to manipulate the opinion of the general public without introducing additional treatments for the views of party constituents on the matter. This kept our design simple, and empirical evidence shows that the majority of the general public and any given party electorate are aligned in the vast majority of cases (Romeijn, 2020). Mainstream parties (to which the vast majority of our respondents belong) also tend to react to shifts in mean voter positions (Ezrow *et al*., 2011).

7 Because we can check respondents not receiving a treatment but still expressing the belief that they had in a subsequent manipulation check, we can also get a sense of how realistic the treatment is. We can look at the expected positions of these groups among respondents not treated with an interest group position but who (falsely) identified having seen a position in the subsequent manipulation checks. We report the 95 per cent confidence intervals. Without information, 58–83 per cent of respondents guessed that environmental groups were in favour the placement of windmills. 21–53 per cent of respondents guessed that business groups were in favour of placement. A large share of respondents thus thought both types of positions were possible for both group types on the issue. See Appendix A4 for more information.

8 The pre-registration included three additional hypotheses regarding representativeness of the groups and personal ties to groups. These are discussed in paragraph 4.2 and in detail on Appendix A6.

9 The latter is measured by the survey question: ‘In political matters, people often talk of the left and the right. On this scale from 0 to 10, where 0 means left and 10 means right, which number best describes your position?’.

10 The statistical literature notes the utility of covariate adjustment (Lin, 2013) to the extent that covariate adjustment can improve precision – even when randomisation of units to experimental conditions is correctly implemented (Lin, 2013). We therefore included a measure of prior agreement in a general battery of policy questions early on in the survey and prior to the experiment, indicating the extent to which respondents agreed with the statement ‘More windfarms on land should be allowed’, on a five-point scale. For full transparency, we include models both with and without covariate adjustment in all analyses (in the paper the even numbered models have this covariate and the odd-numbered do not). In the text, we highlight when these models show different results, but mainly when it comes to our core results, they are robust to their inclusion.

11 There is a lack of consensus in the methodological literature on how to implement manipulation checks (Kane & Barabas, 2019; Mutz, 2021). Kane and Barabas (2019) argue that including one or more factual questions about the manipulation after the stimulus and the measurement of the dependent variable and dropping those who answer the question incorrectly from the analysis, is advisable to ensure that the respondents have actually been attentive to the manipulation. Mutz (2021), in contrast, opposes dropping respondents from experiments, as it affects the extent to which the conditions are truly randomly assigned. While we focus on effects for all respondents, we have also included models with only those that passed factual manipulation checks in the paper for full transparency. This way readers can assess the influence of this choice. In the text, we highlight when these models show different results. For the effect of public opinion and the direct and conditional effect of environment groups, our results are robust to excluding those respondents that did not pass all manipulation checks. For business groups, evidence of politician responsiveness to their views is stronger in the sample of respondents passing the manipulation checks than in our more careful interpretation of the results based on the full sample.

12 Without information on voters’ positions in the control scenario, the likelihood that respondents favour placement is 51 per cent. This is not significantly different from the cases where voters oppose placing windmills.

13 Panel 3 in Figure 2 presents a potentially curious finding: when environmental groups are against the placement of windmills, the most right-wing politicians are more likely to favour windmills than when the environmental groups actually favour them. In Table A19 in the Appendix, we show that this effect is no longer significant if we include a non-linear effect of ideology. We want to thank one of the anonymous reviewers for proposing this explanation.

14 The lack of an effect for public opinion may stem from the fact that the decision to install a windfarm in a given country as a whole is less salient than the decision to place a windfarm in a specific municipality. In the latter scenario, municipal politicians might be more likely to be kept directly accountable for such a decision.

15 As an indirect test of variation in responsiveness to general public opinion and constituents, we conducted an exploratory test of whether responsiveness to general public opinion varied subject to the extremity of the ideology of the elected representatives in our study. It did not find evidence that centrist legislators inhibit a greater tendency than extremist ones to respond to general public opinion, which we might expect if legislators were primarily responding to citizens with whom they are ideologically aligned. Yet, this test does not rule out that we could have found overall variation in responsiveness towards the opinion of the general public and party constituents with an alternative design.
