# Supplementary material for: Responding to whom? An experimental study of the dynamics of responsiveness to interest groups and the public
Source: J Eur Public Policy. 2024 Feb 7;32(3):727–54. doi: 10.1080/13501763.2024.2306226 (PMC11835311; doi:10.1080/13501763.2024.2306226)
Supplement: Supplemental Material [file RJPP_A_2306226_SM5063.docx]

**Appendix**

| **Table of Contents** | | | |
| --- | --- | --- | --- |
| Appendix A1: Structure of the Sample | | p.2 |  |
|  | Table A1: Response Rates | p.2 |  |
|  | Table A2: Respondent characteristics in population and sample | p.2 |  |
| Appendix A2: Descriptives | | p.3 |  |
|  | Table A3: Descriptives of interval variables | p.3 |  |
|  | Table A4: Descriptives of categorical variables | p.4 |  |
| Appendix A3: Balance Tests | | p.5 |  |
|  | Table A5: Balance Tests | p.5 |  |
| Appendix A4: Respondents’ assumption of support | | p.6 |  |
|  | Table A6: Respondents’ assumption of support | p.6 |  |
| Appendix A5: Alternative Regressions | | p.7 |  |
|  | Table A7: Additional regression models 1: multilevel model | p.7 |  |
|  | Table A8: Additional regression models 2: Netherlands only | p.8 |  |
|  | Table A9: Additional regression models 3: Denmark only | p.9 |  |
|  | Table A10: Additional regression models 4: Municipal data only | p.13 |  |
|  | Table A11: Additional regression models 5: Non-municipal data only | p.14 |  |
|  | Table A12: Additional regression models 6: Public opinion only | p.17 |  |
|  | Table A13: Additional regression models 7: Environmental groups only | p.18 |  |
|  | Table A14: Additional regression models 8: Business groups only | p.19 |  |
|  | Table A15: Additional regression models 9: Public opinion-ideology interaction 1 | p.20 |  |
|  | Table A16: Additional regression models 10: Public opinion-ideology interaction 2 | p.22 |  |
|  | Table A17: Additional regression models 11: Dichotomized public opinion | p.23 |  |
|  | Table A18: Additional regression models 12: Public opinion-interest group interaction | p.25 |  |
|  | Table A19: Additional regression models 13: Squared effect of the left-right dimension | p.27 |  |
| Appendix A6: Additional pre-registered tests | | p.30 |  |
|  | Table A20: Analyses including the representativeness of groups | p.31 |  |
|  | Table A21: Analyses including the membership of business and environmental groups 1 | p.35 |  |
|  | Table A22: Analyses including the membership of business and environmental groups 2 | p.36 |  |
|  | Table A23: Analyses including engagement in business and environmental groups 1 | p.37 |  |
|  | Table A24: Analyses including engagement in business and environmental groups 2 | p.38 |  |

**Appendix A1: Sample Structure**

| *Table A1: Response Rates* | | | | | | | |
| --- | --- | --- | --- | --- | --- | --- | --- |
| **Country** | **Level** | **Emails**  **sent^a^** | **Actual**  **sample^b^** | **Responses ^c^** | **Updated**  **sample ^d^** | **Response**  **rate ^e^** | **Share of sample^f^** |
| Danish | Municipal | 2523 | 2448 | 741 | 2448 | 30.3% | 24.7% |
|  | Regional | 230 | 206 | 84 | 208 | 40.4% | 2.8% |
|  | National | 182 | 175 | 41 | 176 | 23.3% | 1.4% |
|  | Total | 2935 | 2829 | 866 | 2832 | 30.6% | 28.9% |
| Dutch | Municipal | 8538 | 8333 | 1915 | 8338 | 23.0% | 64.0% |
|  | Regional | 578 | 569 | 194 | 569 | 34.1% | 6.5% |
|  | National | 150 | 150 | 19 | 150 | 12.7% | 0.6% |
|  | Total | 9266 | 9052 | 2128 | 9057 | 23.5% | 71.1% |
| Total | Municipal | 11061 | 10781 | 2656 | 10786 | 24.6% | 88.7% |
|  | Regional | 808 | 775 | 278 | 777 | 35.7% | 9.3% |
|  | National | 332 | 325 | 60 | 326 | 18.4% | 2.0% |
|  | Total | 12201 | 11881 | 2994 | 11889 | 25.2% | 100% |

^a^ Emails sent out

^b^ Emails sent by the system minus

1. those who did not receive an email and where no replacement was found

2. those who did not receive an email and where a replacement was found

3. those who did receive an email and indicated that they were no longer a member

^c^ Responses in the system that were matched to email addresses

^d^ Actual surveyed plus those who were excluded between the time the emails were sent out and the creation of the actual sample but *did* answer

^e^ Responses divided by the updated sample

^f^ Responses divided by the total number of responses

| *Table A2: Respondent characteristics in population and sample* | | | |  |
| --- | --- | --- | --- | --- |
| **Characteristic** | **Subcharacteristic** | **Population** | **Responses** | |
| Party Family | Christian-Democratic/Conservative | 20% | 18% | |
|  | Green | 6% | 7% | |
|  | Independent from national parties^a^ | 26% | 24% | |
|  | Liberal | 24% | 24% | |
|  | Radical Right | 4% | 4% | |
|  | Social-Democratic | 14% | 16% | |
|  | Radical Left | 5% | 7% | |
|  | Special Interest | 1% | 1% | |
| Gender | Female | 32% | 29% | |
|  | Male | 68% | 71% | |

^a^ Independent local parties and politicians split off from the PPG.

**Appendix A2: Descriptives**

| *Table A3: Descriptives of interval variables* | | | | | | | | |
| --- | --- | --- | --- | --- | --- | --- | --- | --- |
| **Sample** | **Variable** | **Mean** | **Median** | **Min.** | **Max.** | **S.D.** | **N** |  |
| All | Chance of favouring windfarms | 52.58 | 60.00 | 0 | 100 | 30.75 | 2067 |  |
|  | Prior agreement | 3.27 | 4.00 | 1 | 5 | 1.32 | 2631 |  |
|  | Left-Right self-placement | 5.00 | 5.00 | 0 | 10 | 2.31 | 1927 |  |
|  | Extremism | 1.89 | 2.00 | 0 | 5 | 1.33 | 1927 |  |
|  | Left-Right self-placement-squared | 30.32 | 25.00 | 0 | 100 | 22.94 | 1927 |  |
|  | Environmental Group Linkage | 0.58 | - | 0 | 1 | - | 1866 |  |
|  | Environmental Group Member | 0.25 | - | 0 | 1 | - | 1866 |  |
|  | Business Group Linkage | 0.39 | - | 0 | 1 | - | 1851 |  |
|  | Business Group Member | 0.11 | - | 0 | 1 | - | 1851 |  |
| MC | Chance of favouring windfarms | 53.77 | 60.00 | 0 | 100 | 30.83 | 1290 |  |
|  | Prior agreement | 3.29 | 4.00 | 1 | 5 | 1.33 | 1282 |  |
|  | Left-Right self-placement | 4.93 | 5.00 | 0 | 10 | 2.37 | 1230 |  |
|  | Extremism | 1.96 | 2.00 | 0 | 5 | 1.33 | 1230 |  |
|  | Left-Right self-placement-squared | 29.91 | 25.00 | 0 | 100 | 23.30 | 1230 |  |
|  | Environmental Group Linkage | 0.58 | - | 0 | 1 | - | 1208 |  |
|  | Environmental Group Member | 0.27 | - | 0 | 1 | - | 1208 |  |
|  | Business Group Linkage | 0.36 | - | 0 | 1 | - | 1197 |  |
|  | Business Group Member | 0.11 | - | 0 | 1 | - | 1197 |  |

| *Table A4: Descriptives of categorical variables* | | | |
| --- | --- | --- | --- |
| **Variable** | | **All** | **MC** |
| Voters | |  |  |
|  | 35 | 21% | 22% |
|  | 45 | 19% | 22% |
|  | 55 | 20% | 20% |
|  | 65 | 20% | 21% |
|  | No information | 20% | 14% |
|  | N | 2116 | 1290 |
| Environmental Groups | |  |  |
|  | Against | 44% | 46% |
|  | In favour | 42% | 42% |
|  | No information | 14% | 12% |
|  | Against & 5% | 15% | 15% |
|  | Against & 10% | 14% | 14% |
|  | Against & 25% | 14% | 14% |
|  | In favour & 5% | 14% | 14% |
|  | In favour & 10% | 14% | 13% |
|  | In favour & 25% | 13% | 14% |
|  | N | 2116 | 1290 |
| Business Groups | |  |  |
|  | Against | 43% | 44% |
|  | In favour | 43% | 40% |
|  | No information | 14% | 16% |
|  | Against & 5% | 15% | 16% |
|  | Against & 10% | 14% | 14% |
|  | Against & 25% | 14% | 15% |
|  | In favour & 5% | 13% | 13% |
|  | In favour & 10% | 15% | 14% |
|  | In favour & 25% | 14% | 13% |
|  | N | 2116 | 1290 |
| Sample | |  |  |
|  | Danish Municipal | 25% | 22% |
|  | Danish National | 1% | 1% |
|  | Danish Regional | 3% | 3% |
|  | Dutch Municipal | 64% | 66% |
|  | Dutch National | <1% | <1% |
|  | Dutch Regional | 6% | 7% |
| N | | 2994 | 1290 |

**Appendix A3: Balance Tests**

| *Table A5: Balance Tests* | | | | | | | | | |
| --- | --- | --- | --- | --- | --- | --- | --- | --- | --- |
| **Model** | **A1** | **A2** | **A3** | **A4** | **A5** | **A6** | **A7** | **A8** |  |
| **Dependent Variable** | **Gender = Male** | **Age** | **Left Right** | **Windmill** | **Education = BA/MA** | **Country = NL** | **Level = Municipal** | **Level = Regional** |  |
| **Model** | **Logistic** | **Linear** | **Linear** | **Linear** | **Logistic** | **Logistic** | **Logistic** | **Logistic** |  |
| Intercept | 0.88*** | 56.07*** | 4.98*** | 3.33*** | 0.69*** | 0.69*** | -2.55*** | -3.98*** |  |
|  | (0.19) | (1.11) | (0.21) | (0.12) | (0.20) | (0.19) | (0.31) | (0.69) |  |
| Voters = |  |  |  |  |  |  |  |  |  |
| 45 | 0.01 | -0.96 | 0.05 | 0.04 | 0.07 | 0.21 | 0.37 | -0.70 |  |
|  | (0.16) | (0.87) | (0.17) | (0.09) | (0.15) | (0.15) | (0.24) | (0.62) |  |
| 55 | -0.31** | -0.09 | -0.07 | 0.05 | 0.16 | 0.16 | 0.17 | -0.08 |  |
|  | (0.16) | (0.87) | (0.17) | (0.09) | (0.16) | (0.15) | (0.24) | (0.53) |  |
| 65 | -0.23 | -0.28 | 0.01 | 0.00 | 0.18 | 0.10 | 0.38 | -0.00 |  |
|  | (0.16) | (0.88) | (0.17) | (0.09) | (0.16) | (0.15) | (0.24) | (0.51) |  |
| No information | -0.26* | -0.54 | 0.03 | 0.06 | 0.41** | 0.41*** | 0.03 | -0.33 |  |
|  | (0.16) | (0.87) | (0.17) | (0.09) | (0.16) | (0.16) | (0.25) | (0.55) |  |
| Environmental Groups = |  |  |  |  |  |  |  |  |  |
| Against & 10% | 0.22 | -0.28 | 0.32 | -0.19* | 0.18 | 0.16 | 0.20 | 0.13 |  |
|  | (0.18) | (1.03) | (0.20) | (0.11) | (0.19) | (0.18) | (0.28) | (0.64) |  |
| Against & 25% | 0.12 | -0.11 | -0.07 | -0.11 | 0.06 | -0.01 | -0.03 | -0.52 |  |
|  | (0.18) | (1.02) | (0.20) | (0.11) | (0.19) | (0.18) | (0.29) | (0.74) |  |
| In favour & 5% | 0.20 | 0.95 | 0.24 | -0.12 | -0.01 | 0.13 | 0.26 | -0.38 |  |
|  | (0.18) | (1.02) | (0.20) | (0.11) | (0.18) | (0.18) | (0.28) | (0.74) |  |
| In favour & 10% | 0.19 | 0.59 | 0.33* | -0.22** | -0.11 | 0.06 | 0.42 | -0.15 |  |
|  | (0.18) | (1.02) | (0.19) | (0.11) | (0.18) | (0.18) | (0.27) | (0.68) |  |
| In favour & 25% | 0.40** | -0.44 | 0.10 | -0.18* | 0.23 | 0.23 | -0.35 | 0.56 |  |
|  | (0.19) | (1.03) | (0.20) | (0.11) | (0.19) | (0.19) | (0.32) | (0.60) |  |
| No information | 0.36** | 0.11 | 0.21 | -0.04 | 0.25 | 0.08 | 0.29 | 0.20 |  |
|  | (0.18) | (1.02) | (0.19) | (0.11) | (0.19) | (0.18) | (0.27) | (0.62) |  |
| Business Groups = |  |  |  |  |  |  |  |  |  |
| Against & 10% | 0.11 | -0.20 | -0.33* | 0.08 | 0.02 | 0.09 | 0.08 | -1.55 |  |
|  | (0.18) | (1.04) | (0.20) | (0.11) | (0.19) | (0.18) | (0.27) | (1.10) |  |
| Against & 25% | 0.11 | 0.72 | -0.28 | 0.02 | 0.27 | 0.10 | -0.14 | 0.26 |  |
|  | (0.18) | (1.04) | (0.20) | (0.11) | (0.19) | (0.18) | (0.28) | (0.61) |  |
| In favour & 5% | 0.23 | 2.11** | 0.02 | 0.01 | -0.21 | -0.13 | -0.32 | -0.81 |  |
|  | (0.19) | (1.05) | (0.20) | (0.11) | (0.19) | (0.18) | (0.30) | (0.84) |  |
| In favour & 10% | 0.16 | 1.02 | -0.28 | 0.05 | -0.09 | 0.20 | 0.13 | -0.51 |  |
|  | (0.18) | (1.02) | (0.19) | (0.10) | (0.18) | (0.18) | (0.26) | (0.74) |  |
| In favour & 25% | 0.01 | 0.71 | -0.06 | 0.11 | 0.02 | 0.17 | -0.03 | -0.50 |  |
|  | (0.18) | (1.03) | (0.20) | (0.11) | (0.19) | (0.18) | (0.27) | (0.74) |  |
| No information | 0.07 | -0.91 | -0.08 | 0.02 | 0.04 | 0.21 | -0.09 | 1.10** |  |
|  | (0.18) | (1.03) | (0.19) | (0.11) | (0.19) | (0.19) | (0.28) | (0.54) |  |
| AIC | 2461 | 14465 | 8720 | 7134 | 2336 | 2449 | 1341 | 350 |  |
| R-Squared |  | 0.01 | 0.01 | 0.00 |  |  |  |  |  |
| N | 2116 | 1853 | 1927 | 2094 | 1950 | 2116 | 2116 | 2116 |  |

Note: 0.1 > * > 0.05 > ** > 0.01 > ***

To check whether the random assignment of attribute values produced balanced groups, we regress key respondent characteristics such as self-identified gender, age etc. on the attributes. Table A5 shows that the randomization was generally successful. Out of all the examined relationships between respondent background characteristics and assignment of the different treatments, very few are significant: 13 of the 128 co-efficients are significant (at the 0.1-level or lower), which is exactly what one would expect by chance (0.1 x 128 = 12.8).

**Appendix A4: Respondents’ assumptions of support**

| *Table A6: Respondents’ assumptions of support* | | | |
| --- | --- | --- | --- |
| **Category** |  | **Share** |  |
| Public opinion | 35% | 51% (43-59%) |  |
|  | 45% | 10% (5-15%) |  |
|  | 55% | 13% (8-19%) |  |
|  | 65% | 26% (18-33%) |  |
| N |  | 141 |  |
| Environmental groups | Against | 30% (17-42%) |  |
|  | In favour | 70% (58-83%) |  |
| N |  | 54 |  |
| Business groups | Against | 37% (47-79%) |  |
|  | In favour | 63% (21-53%) |  |
| N |  | 35 |  |
| Share of respondents who (a) did not receive information about support of these groups (b) did not indicate that they did not know or did not receive information, with 95% confidence interval. | | |  |

To show the credibility of varying the positions of the group types on support for windmills, we can look at the assumptions respondents made about these if they did not get treated with an interest position but subsequently (falsely) identified having seen a position in the subsequent manipulation checks. Without information, 30% of respondents guessed that environmental groups were against the placement of windmills (and 70% guessed that they were in favour). 37% of respondents guessed that business groups were against placement (and 63% guessed that they were in favour). A large share of respondents thus thought both types of positions were possible for both group types on the issue.

**Appendix A5: Alternative Regressions**

| *Table A7: Additional regression models 1: multilevel model* | | | | | | |  | |  | |  | |  | |
| --- | --- | --- | --- | --- | --- | --- | --- | --- | --- | --- | --- | --- | --- | --- |
| **Model** | | **A9** | **A10** | **A11** | **A12** | **A13** | | **A14** | | **A15** | | **A16** | |  |
| **Sample** | | **All** | **All** | **MC** | **MC** | **All** | | **All** | | **MC** | | **MC** | |  |
| Intercept | | 65.97*** | 9.09*** | 67.57*** | 10.65*** | 61.97*** | | 4.89 | | 64.71*** | | 7.51* | |  |
|  | | (2.27) | (2.79) | (2.84) | (3.42) | (3.22) | | (3.34) | | (3.74) | | (4.00) | |  |
| Prior agreement | |  | 13.76*** |  | 13.39*** |  | | 13.75*** | |  | | 13.35*** | |  |
|  |  |  | (0.43) |  | (0.54) |  | | (0.43) | |  | | (0.54) | |  |
| Left-Right | | -4.01*** | -1.39*** | -4.38*** | -1.69*** | -3.21*** | | -0.55 | | -3.90*** | | -1.08** | |  |
|  | | (0.29) | (0.25) | (0.34) | (0.30) | (0.54) | | (0.45) | | (0.64) | | (0.53) | |  |
| Voters^a^ = | |  |  |  |  |  | |  | |  | |  | |  |
|  | 45 | 3.97* | 3.67** | 2.98 | 3.81* | 4.29** | | 3.96** | | 3.48 | | 4.28** | |  |
|  |  | (2.07) | (1.68) | (2.43) | (1.99) | (2.07) | | (1.67) | | (2.42) | | (1.98) | |  |
|  | 55 | 7.30*** | 7.12*** | 8.36*** | 8.97*** | 7.66*** | | 7.45*** | | 8.81*** | | 9.45*** | |  |
|  |  | (2.08) | (1.69) | (2.49) | (2.04) | (2.07) | | (1.68) | | (2.47) | | (2.02) | |  |
|  | 65 | 10.49*** | 11.40*** | 10.31*** | 10.61*** | 10.55*** | | 11.48*** | | 10.64*** | | 10.96*** | |  |
|  |  | (2.09) | (1.69) | (2.46) | (2.02) | (2.08) | | (1.69) | | (2.44) | | (2.00) | |  |
|  | No information | 3.51* | 2.99* | 1.13 | 2.33 | 3.44* | | 2.95* | | 0.92 | | 2.23 | |  |
|  |  | (2.07) | (1.68) | (2.76) | (2.26) | (2.07) | | (1.67) | | (2.74) | | (2.24) | |  |
| Environmental Groups^b^ = | |  |  |  |  |  | |  | |  | |  | |  |
|  | In favour | 1.93 | 2.88** | 2.39 | 3.69*** | 13.33*** | | 13.71*** | | 15.69*** | | 16.98*** | |  |
|  |  | (1.44) | (1.17) | (1.74) | (1.43) | (3.38) | | (2.73) | | (3.97) | | (3.24) | |  |
|  | No information | 2.90 | 1.84 | 0.68 | 1.69 | 6.42 | | 5.95 | | 15.31** | | 13.12*** | |  |
|  |  | (2.01) | (1.63) | (2.62) | (2.15) | (5.02) | | (4.05) | | (6.24) | | (5.09) | |  |
|  | In favour * Left-Right |  |  |  |  | -2.28*** | | -2.16*** | | -2.63*** | | -2.64*** | |  |
|  |  |  |  |  |  | (0.61) | | (0.50) | | (0.72) | | (0.59) | |  |
|  | No information * Left-Right |  |  |  |  | -0.72 | | -0.84 | | -3.03** | | -2.37** | |  |
|  |  |  |  |  |  | (0.92) | | (0.74) | | (1.19) | | (0.98) | |  |
| Business Groups^c^ = | |  |  |  |  |  | |  | |  | |  | |  |
|  | In favour | 1.65 | 1.57 | 4.50** | 3.75** | -0.85 | | -0.51 | | -5.76 | | -5.03 | |  |
|  |  | (1.43) | (1.16) | (1.78) | (1.46) | (3.41) | | (2.76) | | (4.10) | | (3.34) | |  |
|  | No information | -0.86 | -1.48 | 2.71 | 1.40 | -3.69 | | -2.88 | | -2.89 | | -1.65 | |  |
|  |  | (2.01) | (1.64) | (2.35) | (1.92) | (4.78) | | (3.86) | | (5.37) | | (4.38) | |  |
|  | In favour * Left-Right |  |  |  |  | 0.46 | | 0.38 | | 2.01*** | | 1.70*** | |  |
|  |  |  |  |  |  | (0.62) | | (0.50) | | (0.75) | | (0.61) | |  |
|  | No information * Left-Right |  |  |  |  | 0.58 | | 0.30 | | 1.13 | | 0.62 | |  |
|  |  |  |  |  |  | (0.86) | | (0.70) | | (0.98) | | (0.80) | |  |
| Random Intercept | | 0.00  (0.00) | 3.61  (3.58) | 1.75  (8.07) | 4.18  (4.63) | 0.00  (0.00) | | 3.47  (3.45) | | 0.00  (0.00) | | 3.65  (4.21) | |  |
| AIC | | 18412 | 17442 | 11746 | 11178 | 18405 | | 17430 | | 11730 | | 11155 | |  |
| N | | 1921 | 1904 | 1230 | 1222 | 1921 | | 1904 | | 1230 | | 1222 | |  |

Random intercepts per subsample  ^a^ reference group: ‘35’, ^b^ reference group: ‘against’, ^c^ reference group ‘against’. 0.1 > * > 0.05 > ** > 0.01 > ***

| *Table A8: Additional regression models 2: Netherlands only* | | | | | | |  | |  | |  | |  | |
| --- | --- | --- | --- | --- | --- | --- | --- | --- | --- | --- | --- | --- | --- | --- |
| **Model** | | **A17** | **A18** | **A19** | **A20** | **A21** | | **A22** | | **A23** | | **A24** | |  |
| **Sample** | | **All** | **All** | **MC** | **MC** | **All** | | **All** | | **MC** | | **MC** | |  |
| Intercept | | 67.66*** | 11.43*** | 70.28*** | 14.24*** | 63.78*** | | 7.25* | | 66.31*** | | 10.93** | |  |
|  | | (2.58) | (3.13) | (3.02) | (3.94) | (3.66) | | (3.80) | | (4.19) | | (4.59) | |  |
| Prior agreement | |  | 12.53*** |  | 12.02*** |  | | 12.53*** | |  | | 12.01*** | |  |
|  |  |  | (0.50) |  | (0.64) |  | | (0.50) | |  | | (0.63) | |  |
| Left-Right | | -4.37*** | -1.76*** | -4.81*** | -2.10*** | -3.56*** | | -0.89* | | -4.00*** | | -1.41** | |  |
|  | | (0.33) | (0.29) | (0.39) | (0.36) | (0.62) | | (0.53) | | (0.73) | | (0.63) | |  |
| Voters^a^ = | |  |  |  |  |  | |  | |  | |  | |  |
|  | 45 | 5.21** | 6.04*** | 3.46 | 5.80** | 5.39** | | 6.22*** | | 4.03 | | 6.26*** | |  |
|  |  | (2.32) | (1.94) | (2.73) | (2.33) | (2.31) | | (1.94) | | (2.73) | | (2.32) | |  |
|  | 55 | 7.05*** | 7.74*** | 6.97** | 9.36*** | 7.16*** | | 7.88*** | | 7.22*** | | 9.58*** | |  |
|  |  | (2.32) | (1.95) | (2.79) | (2.38) | (2.32) | | (1.94) | | (2.77) | | (2.36) | |  |
|  | 65 | 12.17*** | 13.77*** | 11.12*** | 12.56*** | 12.03*** | | 13.62*** | | 11.29*** | | 12.62*** | |  |
|  |  | (2.35) | (1.97) | (2.75) | (2.34) | (2.35) | | (1.96) | | (2.74) | | (2.32) | |  |
|  | No information | 2.13 | 3.07 | 0.02 | 3.09 | 2.05 | | 2.99 | | -0.01 | | 3.01 | |  |
|  |  | (2.28) | (1.91) | (3.06) | (2.61) | (2.27) | | (1.90) | | (3.04) | | (2.59) | |  |
| Environmental Groups^b^ = | |  |  |  |  |  | |  | |  | |  | |  |
|  | In favour | 0.69 | 1.82 | 0.04 | 1.88 | 10.73*** | | 12.50*** | | 12.12*** | | 14.27*** | |  |
|  |  | (1.60) | (1.34) | (1.93) | (1.64) | (3.81) | | (3.19) | | (4.42) | | (3.75) | |  |
|  | No information | 2.95 | 1.90 | 0.78 | 1.58 | 14.32** | | 10.79** | | 18.96*** | | 15.68*** | |  |
|  |  | (2.22) | (1.87) | (2.93) | (2.51) | (5.76) | | (4.82) | | (7.13) | | (6.06) | |  |
|  | In favour * Left-Right |  |  |  |  | -2.01*** | | -2.14*** | | -2.42*** | | -2.47*** | |  |
|  |  |  |  |  |  | (0.70) | | (0.58) | | (0.82) | | (0.69) | |  |
|  | No information * Left-Right |  |  |  |  | -2.30** | | -1.81** | | -3.82*** | | -2.97** | |  |
|  |  |  |  |  |  | (1.06) | | (0.89) | | (1.37) | | (1.17) | |  |
| Business Groups^c^ = | |  |  |  |  |  | |  | |  | |  | |  |
|  | In favour | 1.76 | 1.83 | 4.55** | 3.86** | -2.18 | | -1.43 | | -4.03 | | -5.35 | |  |
|  |  | (1.59) | (1.33) | (1.99) | (1.70) | (3.88) | | (3.24) | | (4.66) | | (3.95) | |  |
|  | No information | -1.28 | -1.08 | 0.83 | 0.50 | -3.64 | | -3.17 | | -1.30 | | -2.04 | |  |
|  |  | (2.24) | (1.88) | (2.58) | (2.19) | (5.34) | | (4.46) | | (5.83) | | (4.93) | |  |
|  | In favour * Left-Right |  |  |  |  | 0.76 | | 0.61 | | 1.67* | | 1.79** | |  |
|  |  |  |  |  |  | (0.71) | | (0.60) | | (0.86) | | (0.73) | |  |
|  | No information * Left-Right |  |  |  |  | 0.47 | | 0.43 | | 0.39 | | 0.50 | |  |
|  |  |  |  |  |  | (0.97) | | (0.81) | | (1.08) | | (0.91) | |  |
| Sample^d^= | |  |  |  |  |  | |  | |  | |  | |  |
|  | Dutch National | 7.45 | 4.58 | 13.75 | 10.98 | 6.69 | | 3.52 | | 12.86 | | 9.71 | |  |
|  |  | (9.02) | (7.54) | (9.87) | (8.37) | (9.08) | | (7.57) | | (9.85) | | (8.33) | |  |
|  | Dutch Regional | -4.75* | 1.82 | -5.48* | 0.57 | -4.90* | | 1.74 | | -5.62* | | 0.55 | |  |
|  |  | (2.61) | (2.19) | (3.05) | (2.61) | (2.60) | | (2.19) | | (3.04) | | (2.59) | |  |
| R-squared | | 0.13 | 0.40 | 0.17 | 0.41 | 0.14 | | 0.41 | | 0.19 | | 0.42 | |  |
| N | | 1441 | 1431 | 924 | 918 | 1441 | | 1431 | | 924 | | 918 | |  |

^a^ reference group: ‘35’, ^b^ reference group: ‘against’, ^c^ reference group ‘against’, ^d^ reference group ‘Dutch municipal’. 0.1 > * > 0.05 > ** > 0.01 > ***

| *Table A9: Additional regression models 3: Denmark only* | | | | | | |  | |  | |  | |  | |
| --- | --- | --- | --- | --- | --- | --- | --- | --- | --- | --- | --- | --- | --- | --- |
| **Model** | | **A25** | **A26** | **A27** | **A28** | **A29** | | **A30** | | **A31** | | **A32** | |  |
| **Sample** | | **All** | **All** | **MC** | **MC** | **All** | | **All** | | **MC** | | **MC** | |  |
| Intercept | | 61.46*** | -1.16 | 57.91*** | -0.27 | 56.49*** | | -5.02 | | 58.15*** | | -4.02 | |  |
|  | | (4.87) | (4.73) | (5.98) | (5.77) | (6.81) | | (5.92) | | (8.41) | | (7.39) | |  |
| Prior agreement | |  | 17.12*** |  | 16.76*** |  | | 16.99*** | |  | | 16.67*** | |  |
|  |  |  | (0.85) |  | (1.07) |  | | (0.86) | |  | | (1.07) | |  |
| Left-Right | | -3.03*** | -0.51 | -3.06*** | -0.85 | -2.13* | | 0.28 | | -3.42** | | -0.28 | |  |
|  | | (0.60) | (0.46) | (0.74) | (0.57) | (1.11) | | (0.83) | | (1.37) | | (1.04) | |  |
| Voters^a^ = | |  |  |  |  |  | |  | |  | |  | |  |
|  | 45 | 0.35 | -4.47 | 1.32 | -3.22 | 1.09 | | -4.00 | | 2.34 | | -2.26 | |  |
|  |  | (4.54) | (3.33) | (5.29) | (3.92) | (4.54) | | (3.35) | | (5.30) | | (3.92) | |  |
|  | 55 | 8.33* | 5.00 | 14.64*** | 7.54* | 9.22** | | 5.47 | | 17.04*** | | 8.99** | |  |
|  |  | (4.54) | (3.36) | (5.50) | (4.11) | (4.58) | | (3.40) | | (5.56) | | (4.16) | |  |
|  | 65 | 6.56 | 4.40 | 10.31* | 4.92 | 7.14 | | 4.70 | | 11.99** | | 6.37 | |  |
|  |  | (4.50) | (3.31) | (5.40) | (4.02) | (4.52) | | (3.33) | | (5.45) | | (4.06) | |  |
|  | No information | 10.54** | 2.52 | 8.28 | -0.26 | 9.81** | | 2.19 | | 7.86 | | -0.40 | |  |
|  |  | (4.81) | (3.54) | (6.27) | (4.66) | (4.83) | | (3.56) | | (6.25) | | (4.64) | |  |
| Environmental Groups^b^ = | |  |  |  |  |  | |  | |  | |  | |  |
|  | In favour | 6.01* | 5.71** | 11.15*** | 8.47*** | 20.00*** | | 15.24*** | | 26.58*** | | 23.54*** | |  |
|  |  | (3.22) | (2.37) | (4.00) | (2.96) | (7.32) | | (5.39) | | (9.07) | | (6.69) | |  |
|  | No information | 3.01 | 2.58 | 1.31 | 2.45 | -8.02 | | -1.88 | | 11.21 | | 9.89 | |  |
|  |  | (4.57) | (3.35) | (5.72) | (4.23) | (10.41) | | (7.66) | | (13.17) | | (9.72) | |  |
|  | In favour * Left-Right |  |  |  |  | -2.72** | | -1.86* | | -2.99* | | -2.90** | |  |
|  |  |  |  |  |  | (1.28) | | (0.95) | | (1.58) | | (1.17) | |  |
|  | No information * Left-Right |  |  |  |  | 2.26 | | 0.89 | | -1.92 | | -1.51 | |  |
|  |  |  |  |  |  | (1.87) | | (1.38) | | (2.46) | | (1.81) | |  |
| Business Groups^c^ = | |  |  |  |  |  | |  | |  | |  | |  |
|  | In favour | 0.94 | 0.45 | 2.73 | 2.15 | 3.33 | | 1.86 | | -10.73 | | -4.71 | |  |
|  |  | (3.18) | (2.34) | (3.93) | (2.91) | (7.22) | | (5.32) | | (8.93) | | (6.60) | |  |
|  | No information | -0.55 | -4.19 | 8.34 | 2.08 | -2.16 | | -0.19 | | -7.74 | | 1.66 | |  |
|  |  | (4.72) | (3.48) | (5.81) | (4.32) | (11.24) | | (8.26) | | (13.76) | | (10.17) | |  |
|  | In favour * Left-Right |  |  |  |  | -0.46 | | -0.28 | | 2.66* | | 1.33 | |  |
|  |  |  |  |  |  | (1.28) | | (0.95) | | (1.59) | | (1.18) | |  |
|  | No information * Left-Right |  |  |  |  | 0.43 | | -0.67 | | 3.27 | | 0.27 | |  |
|  |  |  |  |  |  | (1.92) | | (1.41) | | (2.37) | | (1.76) | |  |
| Sample^d^= | |  |  |  |  |  | |  | |  | |  | |  |
|  | Danish National | 6.49 | 5.26 | 3.05 | 2.67 | 6.14 | | 5.07 | | 2.07 | | 2.42 | |  |
|  |  | (8.29) | (6.06) | (9.47) | (6.99) | (8.25) | | (6.05) | | (9.47) | | (6.98) | |  |
|  | Danish Regional | 3.73 | -0.37 | 3.19 | 0.15 | 3.60 | | -0.14 | | 3.46 | | 0.64 | |  |
|  |  | (4.57) | (3.37) | (6.04) | (4.47) | (4.59) | | (3.40) | | (6.04) | | (4.46) | |  |
| R-squared | | 0.08 | 0.51 | 0.12 | 0.52 | 0.10 | | 0.51 | | 0.14 | | 0.54 | |  |
| N | | 480 | 473 | 306 | 304 | 480 | | 473 | | 306 | | 304 | |  |

^a^ reference group: ‘35’, ^b^ reference group: ‘against’, ^c^ reference group ‘against’, ^d^ reference group ‘Danish municipal’. 0.1 > * > 0.05 > ** > 0.01 > ***

| *Figure A1: Chance in Favour and Positions of Voters* | *Figure A2: Chance in Favour if Environmental Groups are against* |
| --- | --- |
|  |  |
| Based on model A22 and A30 | Netherlands = black line; Denmark = grey line; based on model A22 and A30 |
| *Figure A3: Chance in Favour if Environmental Groups are in Favour* | *Figure A4: Chance in Favour if Environmental Groups are Neutral* |
|  |  |
| Netherlands = black line; Denmark = grey line; based on model A22 and A30 | Netherlands = black line; Denmark = grey line; based on model A22 and A30 |
|  |  |
|  |  |
|  |  |
|  |  |
| *Figure A5: Chance in Favour if Business is Against* | *Figure A6: Chance in Favour if Business is Neutral* |
|  |  |
| Netherlands = black line; Denmark = grey line; based on model A22 and A30 | Netherlands = black line; Denmark = grey line; based on model A22 and A30 |
| *Figure A7: Chance in Favour if Business is Neutral* |  |
|  |  |
| Netherlands = black line; Denmark = grey line; based on model A22 and A30 |  |

In nearly all cases the estimates from the Dutch-only and Danish-only models are not significantly different from each other. There are two exceptions: when voters are least supportive of placing windmills, Danes are more supportive than Dutch politicians; when environmental groups are in favour, Danish centre and centre-right politicians favour windmills more than Dutch politicians.

| *Table A10: Additional regression models 4: Municipal data only* | | | | | | |  | |  | |  | |  | |
| --- | --- | --- | --- | --- | --- | --- | --- | --- | --- | --- | --- | --- | --- | --- |
| **Model** | | **A33** | **A34** | **A35** | **A36** | **A47** | | **A38** | | **A39** | | **A40** | |  |
| **Sample** | | **All** | **All** | **MC** | **MC** | **All** | | **All** | | **MC** | | **MC** | |  |
| Intercept | | 66.31*** | 11.97*** | 68.16*** | 14.54*** | 62.24*** | | 7.92** | | 65.32*** | | 11.37*** | |  |
|  | | (2.71) | (2.93) | (3.25) | (3.66) | (3.65) | | (3.54) | | (4.30) | | (4.34) | |  |
| Prior agreement | |  | 13.34*** |  | 12.85*** |  | | 13.32*** | |  | | 12.76*** | |  |
|  |  |  | (0.46) |  | (0.59) |  | | (0.46) | |  | | (0.58) | |  |
| Left-Right | | -3.76*** | -1.46*** | -3.99*** | -1.69*** | -2.98*** | | -0.66 | | -3.57*** | | -1.11* | |  |
|  | | (0.31) | (0.27) | (0.37) | (0.33) | (0.58) | | (0.49) | | (0.68) | | (0.58) | |  |
| Voters^a^ = | |  |  |  |  |  | |  | |  | |  | |  |
|  | 45 | 4.41** | 4.08** | 4.53* | 4.83** | 4.78** | | 4.41** | | 5.28** | | 5.49** | |  |
|  |  | (2.19) | (1.80) | (2.57) | (2.14) | (2.19) | | (1.80) | | (2.56) | | (2.13) | |  |
|  | 55 | 8.54*** | 8.59*** | 10.05*** | 11.05*** | 8.88*** | | 8.89*** | | 10.59*** | | 11.52*** | |  |
|  |  | (2.18) | (1.80) | (2.63) | (2.20) | (2.17) | | (1.79) | | (2.61) | | (2.18) | |  |
|  | 65 | 11.44*** | 12.79*** | 11.01*** | 11.65*** | 11.55*** | | 12.91*** | | 11.57*** | | 12.11*** | |  |
|  |  | (2.21) | (1.82) | (2.60) | (2.17) | (2.21) | | (1.81) | | (2.58) | | (2.16) | |  |
|  | No information | 3.71* | 3.70** | 0.47 | 2.67 | 3.69* | | 3.68** | | 0.46 | | 2.62 | |  |
|  |  | (2.17) | (1.79) | (2.91) | (2.43) | (2.17) | | (1.78) | | (2.89) | | (2.41) | |  |
| Environmental Groups^b^ = | |  |  |  |  |  | |  | |  | |  | |  |
|  | In favour | 1.22 | 2.32* | 1.04 | 2.44 | 12.56*** | | 12.82*** | | 14.00*** | | 15.53*** | |  |
|  |  | (1.52) | (1.25) | (1.85) | (1.54) | (3.64) | | (2.99) | | (4.30) | | (3.58) | |  |
|  | No information | 3.28 | 2.34 | -0.00 | 0.98 | 11.02** | | 9.00** | | 17.32*** | | 13.99** | |  |
|  |  | (2.12) | (1.75) | (2.77) | (2.32) | (5.46) | | (4.49) | | (6.69) | | (5.58) | |  |
|  | In favour * Left-Right |  |  |  |  | -2.26*** | | -2.09*** | | -2.56*** | | -2.59*** | |  |
|  |  |  |  |  |  | (0.66) | | (0.54) | | (0.78) | | (0.65) | |  |
|  | No information * Left-Right |  |  |  |  | -1.55 | | -1.34 | | -3.58*** | | -2.69** | |  |
|  |  |  |  |  |  | (0.99) | | (0.82) | | (1.27) | | (1.07) | |  |
| Business Groups^c^ = | |  |  |  |  |  | |  | |  | |  | |  |
|  | In favour | 1.34 | 1.34 | 3.48* | 3.20** | -2.19 | | -1.21 | | -7.64* | | -5.74 | |  |
|  |  | (1.51) | (1.24) | (1.88) | (1.58) | (3.67) | | (3.02) | | (4.42) | | (3.68) | |  |
|  | No information | -0.62 | -1.18 | 2.83 | 1.56 | -4.18 | | -3.26 | | -3.23 | | -1.95 | |  |
|  |  | (2.15) | (1.77) | (2.52) | (2.10) | (5.23) | | (4.29) | | (5.85) | | (4.86) | |  |
|  | In favour * Left-Right |  |  |  |  | 0.67 | | 0.47 | | 2.18*** | | 1.73** | |  |
|  |  |  |  |  |  | (0.67) | | (0.55) | | (0.81) | | (0.67) | |  |
|  | No information * Left-Right |  |  |  |  | 0.73 | | 0.43 | | 1.22 | | 0.72 | |  |
|  |  |  |  |  |  | (0.94) | | (0.78) | | (1.08) | | (0.89) | |  |
| Sample^d^= | |  |  |  |  |  | |  | |  | |  | |  |
|  | Dutch | -2.25 | -4.57*** | -3.06 | -5.04*** | -2.11 | | -4.45*** | | -2.60 | | -4.65*** | |  |
|  |  | (1.65) | (1.36) | (2.02) | (1.69) | (1.64) | | (1.36) | | (2.00) | | (1.67) | |  |
| R-squared | | 0.10 | 0.40 | 0.12 | 0.40 | 0.11 | | 0.40 | | 0.14 | | 0.41 | |  |
| N | | 1711 | 1695 | 1090 | 1082 | 1711 | | 1695 | | 1090 | | 1082 | |  |

^a^ reference group: ‘35’, ^b^ reference group: ‘against’, ^c^ reference group ‘against’, ^d^ reference group ‘Danish municipal’. 0.1 > * > 0.05 > ** > 0.01 > ***

| *Table A11: Additional regression models 5: Non-municipal data only* | | | | | | |  | |  | |  |  |
| --- | --- | --- | --- | --- | --- | --- | --- | --- | --- | --- | --- | --- |
| **Model** | | **A41** | **A42** | **A43** | **A44** | **A45** | | **A46** | | **A47** | | **A48** |
| **Sample** | | **All** | **All** | **MC** | **MC** | **All** | | **All** | | **MC** | | **MC** |
| Intercept | | 83.22*** | 4.53 | 79.00*** | -0.08 | 80.51*** | | -0.92 | | 77.53*** | | -3.63 |
|  | | (10.31) | (8.98) | (11.70) | (10.33) | (11.91) | | (10.04) | | (13.51) | | (11.21) |
| Outcome favourabillity | |  | 17.85*** |  | 17.89*** |  | | 17.88*** | |  | | 18.16*** |
|  |  |  | (1.23) |  | (1.48) |  | | (1.23) | |  | | (1.48) |
| Left-Right | | -5.48*** | -0.12 | -6.43*** | -0.63 | -4.94*** | | 0.91 | | -6.21*** | | -0.12 |
|  | | (0.81) | (0.68) | (0.94) | (0.80) | (1.52) | | (1.14) | | (1.87) | | (1.35) |
| Voters^a^ = | |  |  |  |  |  | |  | |  | |  |
|  | 45 | 1.94 | -1.91 | -3.99 | -3.98 | 1.75 | | -1.94 | | -4.85 | | -5.00 |
|  |  | (6.65) | (4.61) | (7.74) | (5.29) | (6.65) | | (4.59) | | (7.88) | | (5.29) |
|  | 55 | -3.91 | -6.65 | -2.12 | -8.60 | -3.10 | | -5.79 | | -1.58 | | -8.32 |
|  |  | (6.92) | (4.79) | (7.88) | (5.41) | (6.93) | | (4.78) | | (7.99) | | (5.39) |
|  | 65 | 4.97 | -1.81 | 9.78 | 0.89 | 5.00 | | -1.71 | | 9.49 | | 0.63 |
|  |  | (6.83) | (4.78) | (8.13) | (5.61) | (6.85) | | (4.78) | | (8.30) | | (5.61) |
|  | No information | 3.97 | -5.74 | 8.26 | -5.35 | 3.64 | | -5.54 | | 8.15 | | -6.05 |
|  |  | (7.15) | (4.99) | (8.88) | (6.17) | (7.15) | | (4.97) | | (9.04) | | (6.17) |
| Environmental Groups^b^ = | |  |  |  |  |  | |  | |  | |  |
|  | In favour | 6.57 | 4.62 | 12.30** | 10.80*** | 15.28 | | 17.34*** | | 21.44* | | 23.09*** |
|  |  | (4.69) | (3.25) | (5.45) | (3.72) | (9.50) | | (6.59) | | (11.04) | | (7.41) |
|  | No information | -1.73 | -2.16 | 3.03 | 6.63 | -15.83 | | -4.12 | | 1.00 | | 13.36 |
|  |  | (6.46) | (4.48) | (8.12) | (5.56) | (13.21) | | (9.20) | | (18.28) | | (12.30) |
|  | In favour * Left-Right |  |  |  |  | -1.78 | | -2.64** | | -1.85 | | -2.48* |
|  |  |  |  |  |  | (1.73) | | (1.20) | | (2.01) | | (1.35) |
|  | No information * Left-Right |  |  |  |  | 3.25 | | 0.48 | | 0.53 | | -1.46 |
|  |  |  |  |  |  | (2.54) | | (1.77) | | (3.61) | | (2.43) |
| Business Groups^c^ = | |  |  |  |  |  | |  | |  | |  |
|  | In favour | 5.31 | 5.41* | 11.00* | 6.69* | 8.67 | | 4.28 | | 4.81 | | -3.90 |
|  |  | (4.66) | (3.23) | (5.59) | (3.84) | (9.67) | | (6.72) | | (11.69) | | (7.87) |
|  | No information | -2.58 | -3.35 | 2.37 | -0.02 | -6.43 | | -3.78 | | -1.52 | | -1.60 |
|  |  | (6.69) | (4.63) | (7.81) | (5.34) | (13.90) | | (9.60) | | (15.76) | | (10.57) |
|  | In favour * Left-Right |  |  |  |  | -0.86 | | 0.18 | | 1.17 | | 2.10 |
|  |  |  |  |  |  | (1.79) | | (1.24) | | (2.14) | | (1.44) |
|  | No information * Left-Right |  |  |  |  | 0.67 | | 0.13 | | 0.83 | | 0.39 |
|  |  |  |  |  |  | (2.30) | | (1.59) | | (2.66) | | (1.78) |
| Sample^d^= | |  |  |  |  |  | |  | |  | |  |
|  | Danish Regional | -4.73 | -6.53 | -2.24 | -2.87 | -4.83 | | -6.03 | | -1.20 | | -1.43 |
|  |  | (8.62) | (5.98) | (9.73) | (6.65) | (8.68) | | (6.00) | | (9.95) | | (6.67) |
|  | Dutch National | 1.80 | -1.20 | 10.33 | 5.95 | 0.48 | | -3.56 | | 9.01 | | 4.06 |
|  |  | (13.29) | (9.20) | (14.34) | (9.81) | (13.69) | | (9.44) | | (14.89) | | (10.00) |
|  | Dutch Regional | -14.25* | -6.86 | -11.91 | -4.54 | -13.47* | | -6.34 | | -10.86 | | -3.58 |
|  |  | (8.06) | (5.60) | (8.93) | (6.13) | (8.08) | | (5.59) | | (9.16) | | (6.17) |
| R-squared | | 0.24 | 0.64 | 0.37 | 0.71 | 0.26 | | 0.65 | | 0.38 | | 0.72 |
| N | | 210 | 209 | 140 | 140 | 210 | | 209 | | 140 | | 140 |

^a^ reference group: ‘35’, ^b^ reference group: ‘against’, ^c^ reference group ‘against’, ^d^ reference group ‘Danish municipal’. 0.1 > * > 0.05 > ** > 0.01 > ***

| *Figure A8: Chance in Favour and Positions of Voters* | *Figure A9: Chance in Favour if Environmental Groups are against* |
| --- | --- |
|  |  |
| Based on model A38 and A46 | Municipal = black line; Others = grey line; based on model A38 and A46 |
| *Figure A10: Chance in Favour if Environmental Groups are in Favour* | *Figure A11: Chance in Favour if Environmental Groups are Neutral* |
|  |  |
| Municipal = black line; Others = grey line; based on model A38 and A46 | Municipal = black line; Others = grey line; based on model A38 and A46 |
|  |  |
|  |  |
|  |  |
|  |  |
|  |  |
| *Figure A12: Chance in Favour if Business is Against* | *Figure A13: Chance in Favour if Business is Neutral* |
|  |  |
| Municipal = black line; Others = grey line; based on model A38 and A46 | Municipal = black line; Others = grey line; based on model A38 and A46 |
| *Figure A14: Chance in Favour if Business is Neutral* |  |
|  |  |
| Municipal = black line; Others = grey line; based on model A38 and A46 |  |

In nearly all cases, the estimates from the municipal-only and others-only models are not significantly different from each other. The exception is that the estimates for non-municipal politicians are generally more dependent on the left-right dimension than those for municipal politicians, independent of how interest groups position themselves.

| *Table A12: Additional regression models 6: Public opinion only* | | | | | | | | | |
| --- | --- | --- | --- | --- | --- | --- | --- | --- | --- |
| **Model** | | **A49** | **A50** | **A51** | **A52** | **A53** | **A54** | **A55** | **A56** |
| **Data** | | **All** | **All** | **All** | **All** | **MC** | **MC** | **MC** | **MC** |
| Intercept | | 48.74*** | 3.06 | 51.05*** | 4.89*** | 49.24*** | 3.49 | 51.84*** | 5.70** |
|  | | (1.91) | (1.95) | (1.64) | (1.80) | (2.32) | (2.40) | (2.00) | (2.22) |
| Prior agreement | |  | 14.67*** |  | 14.67*** |  | 14.59*** |  | 14.61*** |
|  | |  | (0.40) |  | (0.40) |  | (0.50) |  | (0.50) |
| Voters ^a^ = | |  |  |  |  |  |  |  |  |
| 45 | | 4.85** | 3.85** |  |  | 5.53** | 4.83** |  |  |
|  | | (2.11) | (1.65) |  |  | (2.54) | (1.98) |  |  |
| 55 | | 8.19*** | 7.38*** |  |  | 10.51*** | 9.86*** |  |  |
|  | | (2.11) | (1.64) |  |  | (2.60) | (2.03) |  |  |
| 65 | | 10.91*** | 10.77*** |  |  | 12.43*** | 11.33*** |  |  |
|  | | (2.11) | (1.64) |  |  | (2.57) | (2.00) |  |  |
| In favour | |  |  | 7.20*** | 7.22*** |  |  | 8.75*** | 8.21*** |
|  | |  |  | (1.51) | (1.17) |  |  | (1.83) | (1.43) |
| No information | | 3.59* | 2.85* | 1.25 | 1.00 | 3.55 | 3.53 | 0.79 | 1.12 |
|  | | (2.09) | (1.63) | (1.83) | (1.43) | (2.91) | (2.26) | (2.62) | (2.04) |
|  | |  |  |  |  |  |  |  |  |
| Sample ^b^ = | |  |  |  |  |  |  |  |  |
|  | Danish National | 9.06 | 5.05 | 8.88 | 4.89 | 8.51 | 3.58 | 8.00 | 3.14 |
|  |  | (7.14) | (5.55) | (7.15) | (5.56) | (8.33) | (6.47) | (8.34) | (6.47) |
|  | Danish Regional | 4.40 | 0.08 | 4.40 | 0.05 | 5.14 | 1.04 | 5.25 | 1.15 |
|  |  | (4.02) | (3.15) | (4.03) | (3.15) | (5.46) | (4.24) | (5.46) | (4.24) |
|  | Dutch Municipal | -1.99 | -5.02*** | -1.97 | -5.03*** | -2.42 | -5.31*** | -2.25 | -5.15*** |
|  |  | (1.65) | (1.30) | (1.66) | (1.30) | (2.08) | (1.62) | (2.08) | (1.63) |
|  | Dutch National | 9.10 | -0.50 | 9.29 | -0.31 | 13.21 | 4.98 | 13.35 | 5.10 |
|  |  | (9.75) | (7.58) | (9.77) | (7.59) | (10.93) | (8.48) | (10.94) | (8.50) |
|  | Dutch Regional | -8.11*** | -2.31 | -7.73** | -1.99 | -9.61*** | -3.87 | -9.09** | -3.41 |
|  |  | (3.02) | (2.36) | (3.02) | (2.36) | (3.67) | (2.86) | (3.67) | (2.86) |
| R-squared | | 0.02 | 0.41 | 0.02 | 0.41 | 0.03 | 0.42 | 0.03 | 0.42 |
| N | | 2067 | 2048 | 2067 | 2048 | 1290 | 1282 | 1290 | 1282 |

^a^ reference group: ‘35’, ^b^ reference group ‘Danish municipal’. 0.1 > * > 0.05 > ** > 0.01 > ***

| *Table A13: Additional regression models 7: Environmental groups only* | | | | | | | | | | |
| --- | --- | --- | --- | --- | --- | --- | --- | --- | --- | --- |
| **Model** | | | **A57** | **A58** | **A59** | **A60** | **A61** | **A62** | **A63** | **A64** |
| **Data** | | | **All** | **All** | **MC** | **MC** | **All** | **All** | **MC** | **MC** |
| Intercept | | | 73.31*** | 16.51*** | 76.55*** | 19.60*** | 68.16*** | 11.54*** | 69.23*** | 12.81*** |
|  | | | (2.20) | (2.56) | (2.67) | (3.23) | (2.71) | (2.86) | (3.23) | (3.53) |
| Prior agreement | | |  | 13.71*** |  | 13.40*** |  | 13.70*** |  | 13.38*** |
|  | | |  | (0.44) |  | (0.55) |  | (0.44) |  | (0.55) |
| Left-Right | | | -4.00*** | -1.41*** | -4.36*** | -1.70*** | -2.96*** | -0.40 | -2.92*** | -0.34 |
|  | | | (0.29) | (0.25) | (0.35) | (0.31) | (0.43) | (0.36) | (0.50) | (0.43) |
| Environmental Groups ^a^ = | | |  |  |  |  |  |  |  |  |
|  | | In favour | 1.98 | 2.91** | 2.06 | 3.47** | 13.15*** | 13.58*** | 15.31*** | 16.66*** |
|  | |  | (1.44) | (1.18) | (1.76) | (1.45) | (3.39) | (2.77) | (4.02) | (3.30) |
|  | | No information | 2.59 | 1.51 | 0.56 | 1.56 | 5.74 | 5.13 | 15.34** | 12.90** |
|  | |  | (2.03) | (1.66) | (2.64) | (2.19) | (5.04) | (4.11) | (6.32) | (5.20) |
|  | | In favour * Left-Right |  |  |  |  | -2.24*** | -2.14*** | -2.68*** | -2.67*** |
|  |  |  |  |  |  |  | (0.62) | (0.50) | (0.73) | (0.60) |
|  | | No information * Left-Right |  |  |  |  | -0.65 | -0.74 | -3.07** | -2.36** |
|  |  |  |  |  |  |  | (0.92) | (0.75) | (1.20) | (1.00) |
| Sample ^b^ = | | |  |  |  |  |  |  |  |  |
|  | Danish National | | 7.23 | 5.78 | 5.91 | 4.65 | 7.16 | 5.73 | 6.73 | 5.32 |
|  |  | | (7.45) | (6.07) | (8.51) | (7.00) | (7.43) | (6.04) | (8.46) | (6.94) |
|  | Danish Regional | | 3.03 | 0.78 | 3.97 | 1.99 | 3.26 | 0.96 | 4.45 | 2.42 |
|  |  | | (4.08) | (3.35) | (5.40) | (4.44) | (4.07) | (3.34) | (5.37) | (4.41) |
|  | Dutch Municipal | | -2.33 | -4.66*** | -2.85 | -4.76*** | -2.29 | -4.61*** | -2.48 | -4.45*** |
|  |  | | (1.66) | (1.37) | (2.04) | (1.69) | (1.66) | (1.36) | (2.03) | (1.68) |
|  | Dutch National | | 6.32 | 0.93 | 12.89 | 7.60 | 4.76 | -0.51 | 11.96 | 6.40 |
|  |  | | (9.36) | (7.63) | (10.33) | (8.49) | (9.35) | (7.60) | (10.29) | (8.44) |
|  | Dutch Regional | | -6.74** | -1.78 | -8.05** | -2.96 | -6.62** | -1.68 | -7.88** | -2.77 |
|  |  | | (2.99) | (2.44) | (3.54) | (2.92) | (2.98) | (2.43) | (3.53) | (2.90) |
| R-squared | | | 0.10 | 0.40 | 0.12 | 0.41 | 0.10 | 0.41 | 0.13 | 0.42 |
| N | | | 1921 | 1904 | 1230 | 1222 | 1921 | 1904 | 1230 | 1222 |

^a^ reference group: ‘Against’, ^b^ reference group ‘Danish municipal’. 0.1 > * > 0.05 > ** > 0.01 > ***

| *Table A14: Additional regression models 8: Business groups only* | | | | | | | | | | |
| --- | --- | --- | --- | --- | --- | --- | --- | --- | --- | --- |
| **Model** | | | **A65** | **A66** | **A67** | **A68** | **A69** | **A70** | **A71** | **A72** |
| **Data** | | | **All** | **All** | **MC** | **MC** | **All** | **All** | **MC** | **MC** |
| Intercept | | | 73.71*** | 17.36*** | 75.18*** | 19.72*** | 75.36*** | 18.64*** | 79.87*** | 23.59*** |
|  | | | (2.21) | (2.56) | (2.67) | (3.19) | (2.68) | (2.85) | (3.21) | (3.53) |
| Prior agreement | | |  | 13.68*** |  | 13.32*** |  | 13.68*** |  | 13.29*** |
|  | | |  | (0.44) |  | (0.55) |  | (0.44) |  | (0.55) |
| Left-Right | | | -3.99*** | -1.40*** | -4.38*** | -1.72*** | -4.32*** | -1.66*** | -5.36*** | -2.52*** |
|  | | | (0.29) | (0.25) | (0.35) | (0.31) | (0.43) | (0.36) | (0.51) | (0.44) |
| Business Groups ^a^ = | | |  |  |  |  |  |  |  |  |
|  | | In favour | 1.81 | 1.72 | 4.75*** | 3.95*** | -0.93 | -0.51 | -5.48 | -4.79 |
|  | |  | (1.44) | (1.18) | (1.80) | (1.49) | (3.45) | (2.82) | (4.17) | (3.44) |
|  | | No information | -0.59 | -1.18 | 2.14 | 0.88 | -4.80 | -4.12 | -4.13 | -3.13 |
|  | |  | (2.05) | (1.68) | (2.40) | (1.98) | (4.88) | (3.97) | (5.47) | (4.50) |
|  | | In favour * Left-Right |  |  |  |  | 0.55 | 0.45 | 2.07*** | 1.77*** |
|  |  |  |  |  |  |  | (0.63) | (0.51) | (0.76) | (0.63) |
|  | | No information * Left-Right |  |  |  |  | 0.83 | 0.58 | 1.29 | 0.83 |
|  |  |  |  |  |  |  | (0.87) | (0.71) | (1.00) | (0.82) |
| Sample ^b^ = | | |  |  |  |  |  |  |  |  |
|  | Danish National | | 7.88 | 6.59 | 6.88 | 6.03 | 7.77 | 6.51 | 6.12 | 5.43 |
|  |  | | (7.45) | (6.07) | (8.47) | (6.98) | (7.46) | (6.08) | (8.46) | (6.97) |
|  | Danish Regional | | 3.22 | 0.89 | 3.94 | 2.18 | 3.19 | 0.91 | 4.56 | 2.79 |
|  |  | | (4.08) | (3.35) | (5.39) | (4.44) | (4.09) | (3.36) | (5.38) | (4.44) |
|  | Dutch Municipal | | -2.26 | -4.58*** | -2.65 | -4.48*** | -2.21 | -4.54*** | -2.53 | -4.36*** |
|  |  | | (1.66) | (1.37) | (2.04) | (1.69) | (1.67) | (1.37) | (2.04) | (1.68) |
|  | Dutch National | | 7.53 | 2.65 | 13.03 | 8.51 | 8.21 | 3.10 | 13.35 | 8.65 |
|  |  | | (9.51) | (7.75) | (10.49) | (8.64) | (9.56) | (7.79) | (10.48) | (8.63) |
|  | Dutch Regional | | -6.67** | -1.67 | -8.05** | -2.90 | -6.63** | -1.63 | -7.68** | -2.59 |
|  |  | | (2.99) | (2.45) | (3.54) | (2.92) | (2.99) | (2.45) | (3.53) | (2.92) |
| R-squared | | | 0.10 | 0.40 | 0.13 | 0.41 | 0.10 | 0.40 | 0.13 | 0.41 |
| N | | | 1921 | 1904 | 1230 | 1222 | 1921 | 1904 | 1230 | 1222 |

^a^ reference group: ‘Against’, ^b^ reference group ‘Danish municipal’. 0.1 > * > 0.05 > ** > 0.01 > ***

| *Table A15: Additional regression models 9: Public opinion-ideology interaction 1* | | | | | | | | | | | |  |
| --- | --- | --- | --- | --- | --- | --- | --- | --- | --- | --- | --- | --- |
| **Model** | | **A73** | **A74** | **A75** | **A76** | **A77** | **A78** | **A79** | **A80** | **A81** | **A82** | |
| **Data** | | **All** | **All** | **All** | **All** | **All** | **MC** | **MC** | **MC** | **MC** | **MC** | |
| Intercept | | 68.99*** | 14.07*** | 5.02 | 47.47*** | 2.77 | 70.09*** | 16.41*** | 7.58** | 48.27*** | 4.27 | |
|  | | (3.78) | (3.52) | (3.13) | (2.94) | (2.62) | (4.55) | (4.34) | (3.82) | (3.55) | (3.20) | |
| Prior agreement | |  | 13.79*** | 14.05*** |  | 14.53*** |  | 13.43*** | 13.27*** |  | 14.40*** | |
|  |  |  | (0.43) | (0.87) |  | (0.41) |  | (0.55) | (1.07) |  | (0.52) | |
| Left-Right | | -3.85*** | -1.61*** |  |  |  | -3.82*** | -1.73*** |  |  |  | |
|  | | (0.65) | (0.53) |  |  |  | (0.77) | (0.64) |  |  |  | |
| Extremism | |  |  |  | 0.66 | 0.20 |  |  |  | 0.59 | -0.21 | |
|  | |  |  |  | (1.17) | (0.91) |  |  |  | (1.42) | (1.11) | |
| Voters ^a^ = | |  |  |  |  |  |  |  |  |  |  | |
|  | 45 | 9.74* | 7.66* | 0.18 | 4.89 | 3.18 | 9.81* | 8.03* | -2.75 | 4.30 | 4.31 | |
|  |  | (4.98) | (4.04) | (4.35) | (3.78) | (2.96) | (5.76) | (4.72) | (5.29) | (4.62) | (3.63) | |
|  | 55 | 10.27** | 8.30** | -1.20 | 11.10*** | 9.01*** | 10.45* | 9.49* | 1.38 | 13.98*** | 11.45*** | |
|  |  | (5.06) | (4.10) | (4.43) | (3.78) | (2.95) | (5.93) | (4.86) | (5.49) | (4.73) | (3.70) | |
|  | 65 | 6.13 | 5.32 | 16.75*** | 8.88** | 10.80*** | 10.58* | 7.10 | 14.52*** | 7.50 | 8.73** | |
|  |  | (4.99) | (4.05) | (4.35) | (3.80) | (2.96) | (5.83) | (4.78) | (5.37) | (4.65) | (3.64) | |
|  | No Information | 2.58 | -2.55 | -1.48 | -0.31 | -0.24 | 6.39 | -1.89 | -7.44 | -0.53 | -0.84 | |
|  |  | (5.05) | (4.10) | (4.41) | (3.78) | (2.95) | (6.60) | (5.42) | (5.99) | (5.07) | (3.98) | |
|  | 45 * Left-Right | -1.16 | -0.84 |  |  |  | -1.29 | -0.84 |  |  |  | |
|  |  | (0.90) | (0.73) |  |  |  | (1.04) | (0.85) |  |  |  | |
|  | 55 * Left-Right | -0.58 | -0.20 |  |  |  | -0.30 | 0.00 |  |  |  | |
|  |  | (0.93) | (0.75) |  |  |  | (1.09) | (0.89) |  |  |  | |
|  | 65 * Left-Right | 0.84 | 1.18 |  |  |  | 0.04 | 0.75 |  |  |  | |
|  |  | (0.91) | (0.74) |  |  |  | (1.06) | (0.87) |  |  |  | |
|  | No Information * Left-Right | 0.21 | 1.09 |  |  |  | -0.89 | 0.95 |  |  |  | |
|  |  | (0.92) | (0.74) |  |  |  | (1.20) | (0.99) |  |  |  | |
|  | 45 * Prior agreement |  |  | 1.12 |  |  |  |  | 2.31 |  |  | |
|  |  |  |  | (1.23) |  |  |  |  | (1.49) |  |  | |
|  | 55 * Prior agreement |  |  | 2.59** |  |  |  |  | 2.57* |  |  | |
|  |  |  |  | (1.25) |  |  |  |  | (1.55) |  |  | |
|  | 65 * Prior agreement |  |  | -1.84 |  |  |  |  | -0.94 |  |  | |
|  |  |  |  | (1.24) |  |  |  |  | (1.51) |  |  | |
|  | No Information * Prior agreement |  |  | 1.32 |  |  |  |  | 3.37** |  |  | |
|  |  |  |  | (1.25) |  |  |  |  | (1.71) |  |  | |
|  | 45 * Extremism |  |  |  | -0.61 | 0.07 |  |  |  | -0.29 | -0.10 | |
|  |  |  |  |  | (1.64) | (1.28) |  |  |  | (1.97) | (1.54) | |
|  | 55 * Extremism |  |  |  | -1.90 | -0.87 |  |  |  | -2.22 | -0.82 | |
|  |  |  |  |  | (1.69) | (1.32) |  |  |  | (2.05) | (1.61) | |
|  | 65 * Extremism |  |  |  | 0.68 | 0.21 |  |  |  | 1.84 | 1.15 | |
|  |  |  |  |  | (1.64) | (1.28) |  |  |  | (1.99) | (1.55) | |
|  | No Information * Extremism |  |  |  | 1.97 | 1.60 |  |  |  | 1.51 | 2.03 | |
|  |  |  |  |  | (1.67) | (1.30) |  |  |  | (2.19) | (1.71) | |
| Sample ^b^ = | |  |  |  |  |  |  |  |  |  |  | |
|  | Danish National | 7.09 | 5.56 | 5.18 | 9.96 | 7.28 | 6.50 | 4.81 | 4.38 | 9.53 | 6.66 | |
|  |  | (7.42) | (6.00) | (5.54) | (7.79) | (6.07) | (8.45) | (6.92) | (6.47) | (8.96) | (7.01) | |
|  | Danish Regional | 2.30 | -0.28 | 0.19 | 5.65 | 0.98 | 3.47 | 1.26 | 1.08 | 7.40 | 2.54 | |
|  |  | (4.06) | (3.31) | (3.14) | (4.26) | (3.34) | (5.36) | (4.39) | (4.23) | (5.67) | (4.44) | |
|  | Dutch Municipal | -2.50 | -4.87*** | -4.96*** | -1.15 | -4.34*** | -3.34 | -5.45*** | -4.98*** | -1.42 | -4.65*** | |
|  |  | (1.66) | (1.35) | (1.30) | (1.74) | (1.37) | (2.03) | (1.67) | (1.63) | (2.16) | (1.70) | |
|  | Dutch National | 3.92 | -1.78 | 0.07 | 9.68 | -0.16 | 9.65 | 3.91 | 5.71 | 14.56 | 5.82 | |
|  |  | (9.31) | (7.53) | (7.56) | (9.76) | (7.60) | (10.27) | (8.41) | (8.46) | (10.88) | (8.51) | |
|  | Dutch Regional | -7.34** | -2.01 | -2.21 | -6.87** | -1.75 | -8.81** | -3.64 | -3.79 | -8.39** | -3.14 | |
|  |  | (2.97) | (2.41) | (2.35) | (3.12) | (2.44) | (3.53) | (2.90) | (2.86) | (3.76) | (2.95) | |
| R-squared | | 0.11 | 0.42 | 0.42 | 0.02 | 0.41 | 0.14 | 0.43 | 0.42 | 0.04 | 0.41 | |
| N | | 1922 | 1905 | 2048 | 1922 | 1905 | 1230 | 1222 | 1282 | 1230 | 1222 | |

^a^ reference group: ‘35’, ^b^ reference group ‘Danish municipal’. 0.1 > * > 0.05 > ** > 0.01 > ***

| *Figure A15: Extremism and public opinion* |
| --- |
|  |
| Based on model A77 |
|  |

| *Table A16: Additional regression models 10: Public opinion-ideology interaction 2* | | | | | | | | | | | |
| --- | --- | --- | --- | --- | --- | --- | --- | --- | --- | --- | --- |
| **Model** | | **A83** | **A84** | **A85** | **A86** | **A87** | **A88** | **A89** | **A90** | **A91** | **A92** |
| **Data** | | **All** | **All** | **All** | **All** | **All** | **MC** | **MC** | **MC** | **MC** | **MC** |
| Intercept | | 62.81*** | 7.45* | 8.44** | 64.20*** | 6.62* | 64.01*** | 9.35* | 13.59*** | 67.27*** | 10.53** |
|  | | (4.52) | (4.05) | (4.22) | (4.12) | (3.80) | (5.33) | (4.89) | (5.14) | (4.87) | (4.60) |
| Prior agreement | |  | 13.80*** | 13.16*** |  | 13.74*** |  | 13.41*** | 11.99*** |  | 13.31*** |
|  |  |  | (0.43) | (0.91) |  | (0.43) |  | (0.54) | (1.12) |  | (0.54) |
| Left-Right | | -3.00*** | -0.71 | -0.56 | -3.24*** | -0.55 | -3.34*** | -1.03 | -1.06** | -3.87*** | -1.08** |
|  | | (0.80) | (0.65) | (0.45) | (0.55) | (0.45) | (0.92) | (0.76) | (0.54) | (0.65) | (0.54) |
| Extremism | |  |  |  | -0.21 | -0.04 |  |  |  | -0.28 | -0.44 |
|  | |  |  |  | (1.12) | (0.91) |  |  |  | (1.33) | (1.09) |
| Environmental Groups ^b^ = | | | |  |  |  |  |  |  |  |  |
|  | In favour | 13.47*** | 14.12*** | 13.60*** | 12.61*** | 13.43*** | 15.27*** | 17.09*** | 16.69*** | 14.83*** | 16.49*** |
|  |  | (3.40) | (2.75) | (2.75) | (3.40) | (2.76) | (4.01) | (3.27) | (3.27) | (4.01) | (3.28) |
|  | No information | 6.38 | 5.60 | 5.77 | 6.97 | 6.20 | 15.52** | 12.58** | 13.07** | 16.09** | 13.37*** |
|  |  | (5.04) | (4.07) | (4.07) | (5.04) | (4.08) | (6.30) | (5.15) | (5.13) | (6.29) | (5.15) |
|  | In favour * Left-Right | -2.28*** | -2.22*** | -2.11*** | -2.15*** | -2.12*** | -2.56*** | -2.66*** | -2.56*** | -2.50*** | -2.57*** |
|  |  | (0.62) | (0.50) | (0.50) | (0.62) | (0.50) | (0.73) | (0.60) | (0.60) | (0.73) | (0.60) |
|  | No information * Left-Right | -0.73 | -0.79 | -0.82 | -0.84 | -0.88 | -3.09** | -2.27** | -2.41** | -3.24*** | -2.45** |
|  |  | (0.92) | (0.74) | (0.75) | (0.92) | (0.75) | (1.20) | (0.99) | (0.98) | (1.20) | (0.99) |
| Business Groups ^c^ = | | | |  |  |  |  |  |  |  |  |
|  | In favour | -0.28 | -0.07 | -0.22 | -0.86 | -0.39 | -5.22 | -4.59 | -4.64 | -5.62 | -5.03 |
|  |  | (3.43) | (2.78) | (2.77) | (3.43) | (2.78) | (4.15) | (3.39) | (3.37) | (4.14) | (3.38) |
|  | No information | -3.76 | -2.70 | -2.74 | -3.34 | -2.53 | -3.03 | -1.81 | -1.56 | -2.05 | -1.19 |
|  |  | (4.87) | (3.93) | (3.92) | (4.87) | (3.94) | (5.45) | (4.44) | (4.44) | (5.47) | (4.46) |
|  | In favour * Left-Right | 0.35 | 0.30 | 0.34 | 0.45 | 0.36 | 1.91** | 1.58** | 1.64*** | 1.91** | 1.66*** |
|  |  | (0.62) | (0.51) | (0.50) | (0.62) | (0.51) | (0.76) | (0.62) | (0.62) | (0.76) | (0.62) |
|  | No information * Left-Right | 0.52 | 0.21 | 0.27 | 0.51 | 0.25 | 1.08 | 0.56 | 0.57 | 0.91 | 0.51 |
|  |  | (0.87) | (0.70) | (0.70) | (0.87) | (0.70) | (0.99) | (0.81) | (0.81) | (1.00) | (0.81) |
| Voters ^a^ = | | | |  |  |  |  |  |  |  |  |
|  | 45 | 10.97** | 9.04** | 0.22 | 4.83 | 3.35 | 9.83* | 8.74* | -3.40 | 4.11 | 4.46 |
|  |  | (4.99) | (4.04) | (4.48) | (3.60) | (2.93) | (5.74) | (4.68) | (5.37) | (4.33) | (3.55) |
|  | 55 | 10.24** | 8.31** | -0.92 | 10.97*** | 8.68*** | 9.57 | 9.04* | 0.41 | 13.80*** | 11.06*** |
|  |  | (5.05) | (4.08) | (4.58) | (3.60) | (2.92) | (5.89) | (4.81) | (5.62) | (4.43) | (3.63) |
|  | 65 | 6.78 | 5.85 | 15.94*** | 8.76** | 10.85*** | 12.63** | 9.24* | 12.89** | 8.71** | 9.35*** |
|  |  | (4.99) | (4.03) | (4.48) | (3.62) | (2.93) | (5.80) | (4.74) | (5.50) | (4.35) | (3.55) |
|  | No Information | 2.06 | -3.14 | 0.46 | -0.43 | 0.03 | 6.05 | -2.39 | -6.25 | -1.19 | -0.91 |
|  |  | (5.05) | (4.08) | (4.60) | (3.61) | (2.92) | (6.55) | (5.35) | (6.09) | (4.74) | (3.90) |
|  | 45 * Left-Right | -1.28 | -1.00 |  |  |  | -1.19 | -0.88 |  |  |  |
|  |  | (0.90) | (0.73) |  |  |  | (1.04) | (0.85) |  |  |  |
|  | 55 * Left-Right | -0.52 | -0.18 |  |  |  | -0.11 | 0.09 |  |  |  |
|  |  | (0.93) | (0.75) |  |  |  | (1.08) | (0.88) |  |  |  |
|  | 65 * Left-Right | 0.77 | 1.13 |  |  |  | -0.33 | 0.37 |  |  |  |
|  |  | (0.91) | (0.73) |  |  |  | (1.06) | (0.87) |  |  |  |
|  | No Information * Left-Right | 0.33 | 1.22* |  |  |  | -0.91 | 0.98 |  |  |  |
|  |  | (0.92) | (0.74) |  |  |  | (1.19) | (0.98) |  |  |  |
|  | 45 * Prior agreement |  |  | 1.14 |  |  |  |  | 2.36 |  |  |
|  |  |  |  | (1.26) |  |  |  |  | (1.51) |  |  |
|  | 55 * Prior agreement |  |  | 2.52* |  |  |  |  | 2.72* |  |  |
|  |  |  |  | (1.29) |  |  |  |  | (1.58) |  |  |
|  | 65 * Prior agreement |  |  | -1.41 |  |  |  |  | -0.57 |  |  |
|  |  |  |  | (1.28) |  |  |  |  | (1.54) |  |  |
|  | No Information * Prior agreement |  |  | 0.77 |  |  |  |  | 2.64 |  |  |
|  |  |  |  | (1.30) |  |  |  |  | (1.73) |  |  |
|  | 45 * Extremism |  |  |  | -0.16 | 0.32 |  |  |  | -0.10 | -0.00 |
|  |  |  |  |  | (1.57) | (1.27) |  |  |  | (1.85) | (1.51) |
|  | 55 * Extremism |  |  |  | -1.78 | -0.66 |  |  |  | -2.48 | -0.80 |
|  |  |  |  |  | (1.61) | (1.30) |  |  |  | (1.93) | (1.57) |
|  | 65 * Extremism |  |  |  | 0.95 | 0.31 |  |  |  | 1.13 | 0.87 |
|  |  |  |  |  | (1.57) | (1.27) |  |  |  | (1.86) | (1.52) |
|  | No Information * Extremism |  |  |  | 2.18 | 1.56 |  |  |  | 1.40 | 1.74 |
|  |  |  |  |  | (1.59) | (1.29) |  |  |  | (2.04) | (1.67) |
| Sample ^b^ = | |  |  |  |  |  |  |  |  |  |  |
|  | Danish National | 6.98 | 5.35 | 6.13 | 8.07 | 6.68 | 6.32 | 4.23 | 5.45 | 6.53 | 5.14 |
|  |  | (7.40) | (5.97) | (5.97) | (7.41) | (5.99) | (8.40) | (6.84) | (6.82) | (8.40) | (6.86) |
|  | Danish Regional | 2.13 | -0.40 | 0.42 | 2.17 | 0.16 | 3.97 | 1.57 | 1.87 | 3.46 | 1.59 |
|  |  | (4.07) | (3.31) | (3.30) | (4.07) | (3.32) | (5.33) | (4.34) | (4.34) | (5.32) | (4.35) |
|  | Dutch Municipal | -2.49 | -4.83*** | -4.49*** | -2.15 | -4.42*** | -2.77 | -4.97*** | -4.42*** | -2.62 | -4.62*** |
|  |  | (1.66) | (1.34) | (1.35) | (1.66) | (1.35) | (2.02) | (1.66) | (1.66) | (2.02) | (1.66) |
|  | Dutch National | 4.48 | -1.07 | -0.42 | 4.57 | -0.92 | 9.09 | 3.45 | 4.60 | 9.34 | 4.01 |
|  |  | (9.50) | (7.66) | (7.67) | (9.50) | (7.68) | (10.40) | (8.48) | (8.47) | (10.39) | (8.48) |
|  | Dutch Regional | -6.91** | -1.76 | -1.82 | -6.76** | -1.89 | -8.43** | -3.24 | -3.22 | -8.09** | -3.21 |
|  |  | (2.97) | (2.41) | (2.40) | (2.98) | (2.41) | (3.50) | (2.86) | (2.86) | (3.51) | (2.88) |
| R-squared | | 0.12 | 0.43 | 0.43 | 0.12 | 0.43 | 0.16 | 0.45 | 0.45 | 0.17 | 0.45 |
| N | | 1921 | 1904 | 1904 | 1921 | 1904 | 1230 | 1222 | 1222 | 1230 | 1222 |

^a^ reference group: ‘35’, ^b^ reference group: ‘against’, ^c^ reference group ‘against’, ^d^ reference group ‘Danish municipal’. 0.1 > * > 0.05 > ** > 0.01 > ***

| *Table A17: Additional regression models 11: Dichotomized public opinion* | | | | | |  |
| --- | --- | --- | --- | --- | --- | --- |
| **Model** | | **A93** | **A94** | **A95** | **A96** | |
| **Data** | | **All** | **All** | **MC** | **MC** | |
| Intercept | | 50.01*** | 3.28* | 48.92*** | 2.32 | |
|  | | (1.93) | (1.98) | (2.38) | (2.44) | |
| Prior agreement | |  | 14.68*** |  | 14.64*** | |
|  | |  | (0.40) |  | (0.50) | |
| Voters^a^ = | |  |  |  |  | |
|  | In favour | 7.10*** | 7.06*** | 8.49*** | 7.90*** | |
|  |  | (1.51) | (1.18) | (1.83) | (1.43) | |
|  | No information | 1.27 | 0.97 | 0.58 | 0.81 | |
|  |  | (1.83) | (1.43) | (2.62) | (2.04) | |
| Environmental Groups ^b^ = | |  |  |  |  | |
|  | In favour | 0.91 | 2.26** | 2.07 | 3.62** | |
|  |  | (1.46) | (1.14) | (1.82) | (1.42) | |
|  | No information | 1.59 | 1.09 | 2.02 | 2.76 | |
|  |  | (2.04) | (1.59) | (2.73) | (2.13) | |
| Business Groups ^c^ = | |  |  |  |  | |
|  | In favour | 1.63 | 1.65 | 4.26** | 3.69** | |
|  |  | (1.45) | (1.13) | (1.86) | (1.45) | |
|  | No information | -1.55 | -0.95 | 1.21 | 0.92 | |
|  |  | (2.08) | (1.62) | (2.51) | (1.95) | |
| Sample ^d^ = | |  |  |  |  | |
|  | Danish National | 8.94 | 4.80 | 7.43 | 2.22 | |
|  |  | (7.15) | (5.56) | (8.34) | (6.46) | |
|  | Danish Regional | 4.20 | -0.16 | 4.71 | 0.38 | |
|  |  | (4.03) | (3.16) | (5.47) | (4.24) | |
|  | Dutch Municipal | -2.00 | -5.07*** | -2.14 | -5.10*** | |
|  |  | (1.66) | (1.30) | (2.09) | (1.62) | |
|  | Dutch National | 11.42 | 1.32 | 14.22 | 6.00 | |
|  |  | (9.91) | (7.70) | (11.12) | (8.62) | |
|  | Dutch Regional | -7.37** | -1.85 | -9.13** | -3.46 | |
|  |  | (3.03) | (2.36) | (3.67) | (2.85) | |
| R-squared | | 0.02 | 0.41 | 0.03 | 0.42 | |
| N | | 2066 | 2047 | 1290 | 1282 | |

^a^ reference group: ‘Against’, ^b^ reference group: ‘against’, ^c^ reference group ‘against’, ^d^ reference group ‘Danish municipal’. 0.1 > * > 0.05 > ** > 0.01 > ***

| *Figure A16: Public opinion and interest group effects (No interaction)* |
| --- |
|  |
| Based on model A94 |

| *Table A18: Additional regression models 12: Public opinion-interest group interaction* | | | | | |  |
| --- | --- | --- | --- | --- | --- | --- |
| **Model** | | **A97** | **A98** | **A99** | **A100** | |
| **Data** | | **All** | **All** | **MC** | **MC** | |
| Intercept | | 48.39*** | 2.37 | 45.88*** | 1.27 | |
|  | | (2.37) | (2.24) | (2.82) | (2.68) | |
| Prior agreement | |  | 14.65*** |  | 14.56*** | |
|  | |  | (0.40) |  | (0.50) | |
| Voters^a^ = | |  |  |  |  | |
|  | In favour | 10.77*** | 8.85*** | 14.12*** | 10.69*** | |
|  |  | (2.88) | (2.26) | (3.44) | (2.68) | |
|  | No information | 1.53 | 2.20 | 6.25 | 2.28 | |
|  |  | (3.50) | (2.74) | (5.29) | (4.15) | |
| Environmental Groups ^b^ = | |  |  |  |  | |
|  | In favour | 1.74 | 3.13* | 4.90* | 4.74** | |
|  |  | (2.31) | (1.80) | (2.75) | (2.15) | |
|  | No information | -0.26 | 0.37 | 2.30 | 3.14 | |
|  |  | (3.16) | (2.46) | (3.97) | (3.09) | |
|  | In favour * Voters = In favour | -4.81 | -2.65 | -7.68* | -3.41 | |
|  |  | (3.27) | (2.56) | (3.95) | (3.08) | |
|  | No information * Voters = In favour | 0.94 | 0.22 | -1.28 | -1.58 | |
|  |  | (4.59) | (3.59) | (5.75) | (4.50) | |
|  | In favour * Voters = No information | 5.73 | 0.91 | 1.66 | 1.70 | |
|  |  | (3.98) | (3.11) | (5.56) | (4.34) | |
|  | No information * Voters = No information | 6.81 | 2.38 | -2.94 | -0.38 | |
|  |  | (5.51) | (4.29) | (9.74) | (7.57) | |
| Business Groups ^c^ = | |  |  |  |  | |
|  | In favour | 4.61** | 2.88 | 8.29*** | 5.58** | |
|  |  | (2.27) | (1.77) | (2.78) | (2.17) | |
|  | No information | 0.11 | 0.46 | 3.19 | 1.58 | |
|  |  | (3.37) | (2.62) | (3.74) | (2.90) | |
|  | In favour * Voters = In favour | -3.74 | -1.73 | -5.64 | -3.17 | |
|  |  | (3.24) | (2.53) | (3.99) | (3.12) | |
|  | No information * Voters = In favour | -0.99 | 0.25 | -0.32 | 0.67 | |
|  |  | (4.71) | (3.68) | (5.45) | (4.23) | |
|  | In favour * Voters = No information | -7.34* | -2.52 | -11.27* | -3.55 | |
|  |  | (3.99) | (3.12) | (5.87) | (4.59) | |
|  | No information * Voters = No information | -5.27 | -6.17 | -10.79 | -5.01 | |
|  |  | (5.44) | (4.24) | (7.32) | (5.70) | |
| Sample ^d^ = | |  |  |  |  | |
|  | Danish National | 8.60 | 4.81 | 7.08 | 1.97 | |
|  |  | (7.15) | (5.57) | (8.34) | (6.48) | |
|  | Danish Regional | 4.06 | -0.14 | 4.39 | 0.22 | |
|  |  | (4.03) | (3.16) | (5.46) | (4.24) | |
|  | Dutch Municipal | -1.85 | -4.93*** | -1.98 | -5.04*** | |
|  |  | (1.66) | (1.30) | (2.09) | (1.63) | |
|  | Dutch National | 11.21 | 0.51 | 12.28 | 4.67 | |
|  |  | (9.96) | (7.75) | (11.22) | (8.72) | |
|  | Dutch Regional | -7.41** | -2.00 | -9.59*** | -3.79 | |
|  |  | (3.03) | (2.37) | (3.68) | (2.87) | |
| R-squared | | 0.03 | 0.41 | 0.04 | 0.42 | |
| N | | 2066 | 2047 | 1290 | 1282 | |

^a^ reference group: ‘Against’, ^b^ reference group: ‘against’, ^c^ reference group ‘against’, ^d^ reference group ‘Danish municipal’. 0.1 > * > 0.05 > ** > 0.01 > ***

| *Figure A17: Public opinion-interest group interaction* |
| --- |
|  |
| Based on model A98 |

| *Table A19: Additional regression models 13: Squared effect of the left-right dimension* | | | | | |
| --- | --- | --- | --- | --- | --- |
| **Model** | | **A101** | **A102** | **A103** | **A104** |
| **Sample** | | **All** | **All** | **MC** | **MC** |
| Intercept | | 58.74*** | 2.68 | 57.00*** | 3.79 |
|  | | (4.89) | (4.34) | (5.54) | (5.03) |
| Prior agreement | |  | 13.73*** |  | 13.24*** |
|  |  |  | (0.43) |  | (0.54) |
| Left-Right | | -0.54 | 1.59 | 1.63 | 2.40 |
|  | | (2.04) | (1.65) | (2.32) | (1.90) |
| Left-Right-Squared | | -0.28 | -0.23 | -0.59** | -0.38* |
|  | | (0.21) | (0.17) | (0.24) | (0.20) |
| Voters^a^ = | |  |  |  |  |
|  | 45 | 4.64** | 4.02** | 4.25* | 4.61** |
|  |  | (2.08) | (1.68) | (2.43) | (1.99) |
|  | 55 | 7.69*** | 7.46*** | 9.01*** | 9.49*** |
|  |  | (2.07) | (1.69) | (2.48) | (2.04) |
|  | 65 | 10.53*** | 11.38*** | 10.88*** | 10.98*** |
|  |  | (2.09) | (1.69) | (2.45) | (2.01) |
|  | No information | 3.74* | 2.99* | 1.53 | 2.44 |
|  |  | (2.08) | (1.68) | (2.75) | (2.25) |
| Environmental Groups^b^ = | |  |  |  |  |
|  | In favour | 8.70 | 12.16*** | 12.09** | 14.16*** |
|  |  | (5.32) | (4.30) | (6.13) | (5.01) |
|  | No information | 17.75** | 15.31** | 33.62*** | 26.07*** |
|  |  | (8.40) | (6.80) | (10.89) | (8.92) |
|  | In favour * Left-Right | 0.03 | -1.44 | -1.06 | -1.33 |
|  |  | (2.32) | (1.88) | (2.72) | (2.22) |
|  | No information * Left-Right | -6.57* | -5.74* | -12.92** | -9.40** |
|  |  | (3.72) | (3.02) | (5.01) | (4.12) |
|  | In favour * Left-Right-Squared | -0.22 | -0.07 | -0.14 | -0.12 |
|  |  | (0.23) | (0.19) | (0.28) | (0.23) |
|  | No information * Left-Right-Squared | 0.61 | 0.52* | 1.05** | 0.75* |
|  |  | (0.38) | (0.31) | (0.52) | (0.43) |
|  |  |  |  |  |  |
| Business Groups^c^ = | |  |  |  |  |
|  | In favour | 8.71 | 7.26* | 7.26 | 3.98 |
|  |  | (5.39) | (4.36) | (6.37) | (5.21) |
|  | No information | 0.08 | 1.63 | 7.91 | 5.69 |
|  |  | (7.73) | (6.25) | (8.47) | (6.92) |
|  | In favour * Left-Right | -4.56* | -3.73* | -5.11* | -3.26 |
|  |  | (2.37) | (1.92) | (2.83) | (2.31) |
|  | No information * Left-Right | -1.91 | -2.30 | -5.41 | -3.85 |
|  |  | (3.30) | (2.67) | (3.75) | (3.07) |
|  | In favour * Left-Right-Squared | 0.51** | 0.42** | 0.74** | 0.52** |
|  |  | (0.24) | (0.19) | (0.29) | (0.24) |
|  | No information * Left-Right-Squared | 0.28 | 0.28 | 0.71* | 0.49 |
|  |  | (0.33) | (0.26) | (0.38) | (0.31) |
| Sample^d^= | |  |  |  |  |
|  | Danish National | 7.70 | 6.53 | 7.36 | 5.71 |
|  |  | (7.39) | (5.98) | (8.35) | (6.83) |
|  | Danish Regional | 2.34 | 0.15 | 3.14 | 1.37 |
|  |  | (4.06) | (3.31) | (5.31) | (4.34) |
|  | Dutch Municipal | -2.05 | -4.30*** | -2.52 | -4.50*** |
|  |  | (1.66) | (1.35) | (2.02) | (1.66) |
|  | Dutch National | 5.50 | -0.54 | 9.39 | 4.23 |
|  |  | (9.52) | (7.70) | (10.36) | (8.47) |
|  | Dutch Regional | -6.47** | -1.65 | -7.68** | -2.87 |
|  |  | (2.97) | (2.41) | (3.49) | (2.86) |
| R-squared | | 0.12 | 0.43 | 0.17 | 0.45 |
| N | | 1921 | 1904 | 1230 | 1222 |

^a^ reference group: ‘35’, ^b^ reference group: ‘against’, ^c^ reference group ‘against’, ^d^ reference group ‘Danish municipal’. 0.1 > * > 0.05 > ** > 0.01 > ***

| *Figure A18: Chance in Favour if Environmental Groups are against or no information* | *Figure A19: Chance in Favour if Environmental Groups are in favour or no information* |
| --- | --- |
|  |  |
| Against = black line; Neutral = Grey line; Based on model A102 | In favour = black line; Neutral = Grey line; Based on model A102 |
| *Figure A20: Chance in Favour if Environmental Groups are in favour or against* | *Figure A21: Chance in Favour if Business Groups are against or no information* |
|  |  |
| In favour = black line; Against = Grey line; Based on model A102 | Against = black line; Neutral = Grey line; Based on model A102 |
|  |  |
|  |  |
|  |  |
|  |  |
|  |  |
| *Figure A22: Chance in Favour if Business Groups are in favour or no information* | *Figure A23: Chance in Favour if Business Groups are in favour or against* |
|  |  |
| In favour = black line; Neutral = Grey line; Based on model A102 | In favour = black line; Against = Grey line; Based on model A102 |

**Appendix A6: Additional pre-registered tests**

Our pre-registration included three additional hypotheses. These were not included in the main text of the paper but we include them in the appendix for the sake of transparency and completeness.

The first additional hypothesis we included concerned the representativeness of interest groups. Even if a broad set of interest groups engage in resource exchange with decision-makers as we discussed in the main paper, the “representative potential” of interest groups varies. Representation can be understood as a process of claims making where representatives make claims to represent particular interests (Saward, 2008). For interest groups the challenge is that they are not directly elected but self-authorized representatives. However, provided they are *authorized by* and *accountable to* the affected constituency that they claim to represent, they may be seen as contributing to democratic representation (Montanaro, 2012). Organizations that can claim to affect a substantial share of the people affected by the policy decision should therefore have more leverage in persuading elected representatives. Such groups have the greatest potential to mobilize relevant stakeholders and act as dominant voices within the wider public who ultimately hold politicians accountable for the policies they adopt. They are also likely to play a critical role in securing successful policy implementation. As a result we expect a positive conditioning effect of representativeness on an interest group’s ability to affect representatives, with interest groups more likely to affect the intention of politicians to support a policy proposal, the higher the share of their potential constituency these groups represent.

*A1. Representativeness hypothesis:* The effect of supporting a proposal on the position of politicians is stronger when groups can credibly represent parts of the public affected by a policy.

To assess the effects of the ‘representativeness’ of groups in hypothesis A1, we compare the effect of learning about the varying shares of the relevant constituency that is represented by the interest groups. This was randomly varied in the vignette with Business/Environmental groups claimed to represent 5%/10%/25% of the businesses/citizens in the politician’s municipality/region/country.

| *Table A20: Analyses including the representativeness of groups* | | | | | | | | | |
| --- | --- | --- | --- | --- | --- | --- | --- | --- | --- |
| **Model** | | **A105** | **A106** | **A107** | **A108** | **A109** | **A110** | **A111** | **A112** |
| **Data** | | **All** | **All** | **MC** | **MC** | **All** | **All** | **MC** | **MC** |
| Intercept | | 54.01*** | 5.60*** | 55.22*** | 6.60** | 53.09*** | 7.00*** | 52.54*** | 6.26** |
|  | | (2.13) | (2.13) | (2.67) | (2.67) | (2.15) | (2.11) | (2.72) | (2.65) |
| Prior agreement | |  | 14.75*** |  | 14.75*** |  | 14.66*** |  | 14.64*** |
|  | |  | (0.40) |  | (0.51) |  | (0.40) |  | (0.51) |
| Environmental Groups ^a^ = | |  |  |  |  |  |  |  |  |
|  | Against & 10% | -1.18 | 2.12 | -2.30 | 0.72 |  |  |  |  |
|  |  | (2.51) | (1.96) | (3.12) | (2.43) |  |  |  |  |
|  | Against & 25% | -0.60 | 0.89 | -0.89 | -0.12 |  |  |  |  |
|  |  | (2.48) | (1.94) | (3.08) | (2.40) |  |  |  |  |
|  | In favour & 5% | -1.26 | 0.59 | -0.13 | 1.23 |  |  |  |  |
|  |  | (2.50) | (1.95) | (3.11) | (2.42) |  |  |  |  |
|  | In favour & 10% | 0.04 | 3.51* | 1.08 | 4.56* |  |  |  |  |
|  |  | (2.49) | (1.95) | (3.16) | (2.47) |  |  |  |  |
|  | In favour & 25% | 3.43 | 6.69*** | 2.44 | 6.22** |  |  |  |  |
|  |  | (2.53) | (1.98) | (3.23) | (2.52) |  |  |  |  |
|  | No information | 1.01 | 2.00 | 1.02 | 2.92 |  |  |  |  |
|  |  | (2.49) | (1.95) | (3.27) | (2.55) |  |  |  |  |
| Business Groups ^b^ = | |  |  |  |  |  |  |  |  |
|  | Against & 10% |  |  |  |  | 1.77 | 0.84 | 1.94 | 1.82 |
|  |  |  |  |  |  | (2.52) | (1.98) | (3.17) | (2.47) |
|  | Against & 25% |  |  |  |  | -0.38 | 0.03 | 0.66 | 1.30 |
|  |  |  |  |  |  | (2.49) | (1.95) | (3.10) | (2.41) |
|  | In favour & 5% |  |  |  |  | 0.41 | 1.06 | 0.99 | 0.82 |
|  |  |  |  |  |  | (2.53) | (1.99) | (3.22) | (2.51) |
|  | In favour & 10% |  |  |  |  | 2.86 | 2.73 | 7.56** | 8.53*** |
|  |  |  |  |  |  | (2.45) | (1.93) | (3.16) | (2.47) |
|  | In favour & 25% |  |  |  |  | 3.63 | 2.64 | 7.04** | 4.77* |
|  |  |  |  |  |  | (2.49) | (1.95) | (3.19) | (2.49) |
|  | No information |  |  |  |  | -0.89 | -0.62 | 1.71 | 1.47 |
|  |  |  |  |  |  | (2.51) | (1.97) | (3.05) | (2.38) |
| Sample ^c^ = | |  |  |  |  |  |  |  |  |
|  | Danish National | 7.70 | 3.31 | 7.10 | 1.30 | 8.95 | 4.79 | 7.91 | 3.05 |
|  |  | (7.20) | (5.60) | (8.45) | (6.57) | (7.20) | (5.62) | (8.40) | (6.54) |
|  | Danish Regional | 4.95 | 0.37 | 6.22 | 1.56 | 5.00 | 0.70 | 6.52 | 2.22 |
|  |  | (4.05) | (3.18) | (5.53) | (4.30) | (4.05) | (3.18) | (5.50) | (4.28) |
|  | Dutch Municipal | -2.10 | -5.22*** | -1.95 | -5.00*** | -2.04 | -5.04*** | -1.85 | -4.72*** |
|  |  | (1.67) | (1.31) | (2.10) | (1.64) | (1.66) | (1.31) | (2.10) | (1.64) |
|  | Dutch National | 11.25 | 1.69 | 16.49 | 7.61 | 13.30 | 3.42 | 17.50 | 9.11 |
|  |  | (9.82) | (7.65) | (11.04) | (8.58) | (9.96) | (7.78) | (11.19) | (8.70) |
|  | Dutch Regional | -7.62** | -1.88 | -8.61** | -2.97 | -7.99*** | -2.18 | -9.15** | -3.42 |
|  |  | (3.04) | (2.38) | (3.71) | (2.89) | (3.04) | (2.38) | (3.70) | (2.89) |
| R-squared | | 0.01 | 0.40 | 0.01 | 0.41 | 0.01 | 0.40 | 0.02 | 0.41 |
| N | | 2067 | 2048 | 1290 | 1282 | 2067 | 2048 | 1290 | 1282 |

^a^ reference group: ‘Against & 5%’, ^b^ reference group: ‘Against & 5%’, ^c^ reference group ‘Danish municipal’. 0.1 > * > 0.05 > ** > 0.01 > ***

In Table A20, we find relatively weak and inconsistent results: for environmental groups, we only find a result when we include the prior agreement measure. When the environmental groups are in favour, groups that represent 10% or 25% of the citizens in a given geographical area elicit greater support than when the groups are against. For business groups we only find an effect for those respondents who passed the manipulation check. When they are in favour, business groups that represent 10% or 25% of the business community elicit greater support than when the groups are against. We have visualized this in Figure A24 and A25. Note that although the fifth and sixth column of Figure A24 and the sixth column in Figure A25 are significantly higher than the others, this pattern is not replicated in all analyses.

| *Figure A24: Effect of the representativity of business groups* | *Figure A25: Effect of the representativity of environmental groups* |
| --- | --- |
|  |  |
| Based on model A106 | Based on model A110 |

The second and third additional hypotheses concerned ties between interest groups and politicians. We expected engagement of politicians in a specific type of interest group to make politicians more responsive to learning about the position of the group group towards a policy issue. Given that politicians are boundedly rational, subject to both cognitive and informational constraints (Simon, 1991), they may use engagement in specific types of groups as a shortcut for judging whether to respond to certain policy positions. Drawing upon Social Identity Theory (e.g., Tajfel & Turner, 1986), we can conceptualize engagement within a particular type of interest group as encompassing a collective sense of belonging to the same in-group as the group, leading individuals to exhibit heightened sensitivity towards the views advocated by that interest group. As a result, we would generally expect politicians to be more likely to act in line with the position of a given type of interest group, the stronger they are engaged in the group in question.

*A2. Business tie hypothesis:* Business groups have a greater impact on the positions of politicians the stronger their engagement in business interest groups.

*A3. Civil society tie hypothesis:* Civil society organizations have a greater impact on the positions of politicians the stronger their engagement in civil society groups.

We use information about whether politicians report engagement in environmental groups or business groups, to test hypotheses A2 and A3. Here we examine whether there is a difference in the responsiveness to the positions of a given group type, depending on whether a given politician is engaged in the group type in question.

| *Figure A26: Environmental group positions and links* | *Figure A27: Business group positions and links* |
| --- | --- |
|  |  |
| Based on model A134 | Based on model A134 |

The analyses in Table A21 and A22 include group membership. Table A23 and A24 include a broader form of engagement in these groups (membership, participation in activities, donations, volunteer work or paid work). Both Tables show that politicians who are members of environmental groups are more sensitive to the demands of such groups. This effect, however, disappears once the interactions with left-right ideology are included. Such an effect is not present for members engaged in business groups being more sensitive to the demands of business groups.

| *Table A21: Analyses including the membership of business and environmental groups 1* | | | | | | | | | | | |
| --- | --- | --- | --- | --- | --- | --- | --- | --- | --- | --- | --- |
| **Model** | | | | **A113** | **A114** | **A115** | **A116** | **A117** | **A118** | **A119** | **A120** |
| **Data** | | | | **All** | **All** | **All** | **All** | **All** | **All** | **All** | **All** |
| Intercept | | | | 51.83*** | 7.31*** | 53.28*** | 7.23*** | 51.06*** | 6.17*** | 67.65*** | 12.02*** |
|  | | | | (1.81) | (1.93) | (1.81) | (1.96) | (2.06) | (2.10) | (3.50) | (3.38) |
| Prior agreement | | | |  | 14.51*** |  | 14.60*** |  | 14.54*** |  | 13.76*** |
|  | | | |  | (0.42) |  | (0.43) |  | (0.43) |  | (0.45) |
| Environmental Group Member | | | | 5.61** | -1.24 |  |  | 5.37** | -1.25 | 2.85 | -1.17 |
|  |  |  |  | (2.43) | (1.91) |  |  | (2.47) | (1.95) | (2.44) | (1.99) |
| Business Group Member | | | |  |  | -0.43 | 3.13 | -0.66 | 2.85 | 3.49 | 4.37 |
|  |  |  |  |  |  | (3.40) | (2.66) | (3.44) | (2.69) | (3.36) | (2.72) |
| Left-Right | | | |  |  |  |  |  |  | -3.19*** | -0.68 |
|  | | | |  |  |  |  |  |  | (0.58) | (0.48) |
| Environmental Groups^a^ = | | | |  |  |  |  |  |  |  |  |
|  | In favour | | | -0.18 | 1.22 |  |  | 0.14 | 1.44 | 11.30*** | 11.24*** |
|  |  | | | (1.77) | (1.39) |  |  | (1.80) | (1.41) | (3.90) | (3.17) |
|  | No information | | | 2.94 | 0.75 |  |  | 3.09 | 0.98 | 10.37* | 5.60 |
|  |  | | | (2.46) | (1.94) |  |  | (2.51) | (1.97) | (5.90) | (4.79) |
|  | In favour * Environmental Group Member | | | 8.76** | 8.91*** |  |  | 9.15** | 8.98*** | 4.08 | 5.69* |
|  |  |  |  | (3.55) | (2.78) |  |  | (3.61) | (2.83) | (3.59) | (2.92) |
|  | No information * Environmental Group Member | | | -1.50 | 3.34 |  |  | -1.46 | 3.00 | -6.21 | 0.72 |
|  |  |  |  | (5.06) | (3.97) |  |  | (5.14) | (4.03) | (5.20) | (4.23) |
|  | In favour * Left-Right | | |  |  |  |  |  |  | -1.96*** | -1.82*** |
|  |  |  |  |  |  |  |  |  |  | (0.66) | (0.54) |
|  | No information * Left-Right | | |  |  |  |  |  |  | -1.32 | -0.86 |
|  |  |  |  |  |  |  |  |  |  | (1.02) | (0.83) |
| Business Groups^b^ = | | | |  |  |  |  |  |  |  |  |
|  | | In favour | |  |  | 1.53 | 1.48 | 1.55 | 1.63 | -0.69 | -0.10 |
|  | |  | |  |  | (1.64) | (1.29) | (1.64) | (1.29) | (3.57) | (2.90) |
|  | | No information | |  |  | -1.56 | -1.76 | -1.47 | -1.45 | -3.28 | -1.98 |
|  | |  | |  |  | (2.36) | (1.85) | (2.37) | (1.86) | (5.04) | (4.09) |
|  | | In favour * Business Group Member | |  |  | -2.76 | -2.51 | -3.59 | -3.75 | -4.32 | -4.35 |
|  |  |  |  |  |  | (5.02) | (3.94) | (5.09) | (4.00) | (4.94) | (4.02) |
|  | | No information * Business Group Member | |  |  | 8.22 | 4.82 | 8.48 | 4.90 | 9.27 | 5.18 |
|  |  |  |  |  |  | (6.96) | (5.44) | (7.03) | (5.51) | (6.87) | (5.58) |
|  | | In favour * Left-Right | |  |  |  |  |  |  | 0.51 | 0.37 |
|  | |  |  |  |  |  |  |  |  | (0.66) | (0.53) |
|  | | No information * Left-Right | |  |  |  |  |  |  | 0.43 | 0.15 |
|  |  |  |  |  |  |  |  |  |  | (0.92) | (0.75) |
| Sample^c^ = | | | |  |  |  |  |  |  |  |  |
|  | | | Danish National | 9.61 | 6.77 | 9.01 | 5.15 | 9.89 | 7.33 | 9.84 | 9.49 |
|  | | |  | (7.36) | (5.76) | (7.24) | (5.66) | (7.39) | (5.78) | (7.72) | (6.26) |
|  | | | Danish Regional | 8.93* | 2.43 | 6.50 | 1.74 | 8.00 | 2.61 | 2.07 | 0.83 |
|  | | |  | (4.85) | (3.84) | (4.75) | (3.75) | (5.09) | (4.03) | (5.01) | (4.12) |
|  | | | Dutch Municipal | -2.00 | -4.84*** | -1.13 | -4.38*** | -1.60 | -4.53*** | -2.33 | -4.55*** |
|  | | |  | (1.76) | (1.39) | (1.78) | (1.40) | (1.79) | (1.41) | (1.72) | (1.41) |
|  | | | Dutch National | 12.01 | 1.70 | 13.78 | 4.54 | 14.10 | 4.08 | 7.69 | 1.55 |
|  | | |  | (9.77) | (7.65) | (10.05) | (7.86) | (9.99) | (7.82) | (9.63) | (7.81) |
|  | | | Dutch Regional | -6.85** | -1.85 | -5.68* | -0.88 | -6.93** | -1.53 | -6.98** | -1.71 |
|  | | |  | (3.16) | (2.48) | (3.20) | (2.51) | (3.20) | (2.51) | (3.07) | (2.50) |
| R-squared | | | | 0.03 | 0.41 | 0.01 | 0.40 | 0.03 | 0.41 | 0.11 | 0.42 |
| N | | | | 1860 | 1843 | 1845 | 1830 | 1812 | 1797 | 1794 | 1779 |

^a^ reference group: ‘Against’, ^b^ reference group: ‘Against’, ^c^ reference group ‘Danish municipal’. 0.1 > * > 0.05 > ** > 0.01 > ***

| *Table A22: Analyses including the membership of business and environmental groups 2* | | | | | | | | | | |
| --- | --- | --- | --- | --- | --- | --- | --- | --- | --- | --- |
| **Model** | | | **A121** | **A122** | **A123** | **A124** | **A125** | **A126** | **A127** | **A128** |
| **Data** | | | **MC** | **MC** | **MC** | **MC** | **MC** | **MC** | **MC** | **MC** |
| Intercept | | | 52.56*** | 7.83*** | 52.78*** | 6.84*** | 49.88*** | 5.27** | 70.40*** | 15.01*** |
|  | | | (2.21) | (2.38) | (2.23) | (2.42) | (2.54) | (2.59) | (4.09) | (4.09) |
| Prior agreement | | |  | 14.48*** |  | 14.57*** |  | 14.44*** |  | 13.30*** |
|  | | |  | (0.52) |  | (0.53) |  | (0.53) |  | (0.56) |
| Environmental Group Member | | | 6.48** | 0.15 |  |  | 6.64** | 0.59 | 5.02* | 1.40 |
|  |  |  | (2.86) | (2.25) |  |  | (2.92) | (2.30) | (2.82) | (2.32) |
| Business Group Member | | |  |  | -1.17 | 3.91 | -1.51 | 3.26 | 4.63 | 6.27* |
|  |  |  |  |  | (4.13) | (3.23) | (4.19) | (3.29) | (4.02) | (3.30) |
| Left-Right | | |  |  |  |  |  |  | -4.06*** | -1.35** |
|  | | |  |  |  |  |  |  | (0.67) | (0.56) |
| Environmental Groups ^a^ = | | |  |  |  |  |  |  |  |  |
|  | | In favour | -0.29 | 1.39 |  |  | 0.40 | 1.86 | 11.45** | 13.03*** |
|  | |  | (2.20) | (1.72) |  |  | (2.23) | (1.75) | (4.56) | (3.74) |
|  | | No information | 1.93 | 1.22 |  |  | 2.36 | 1.82 | 19.76*** | 14.78** |
|  | |  | (3.21) | (2.53) |  |  | (3.29) | (2.60) | (7.37) | (6.06) |
|  | | In favour * Environmental Group Member | 8.05* | 8.45** |  |  | 8.06* | 8.19** | 2.38 | 4.21 |
|  |  |  | (4.22) | (3.31) |  |  | (4.30) | (3.37) | (4.18) | (3.44) |
|  | | No information * Environmental Group Member | -0.12 | 3.06 |  |  | -0.28 | 2.35 | -10.81 | -3.95 |
|  |  |  | (6.60) | (5.17) |  |  | (6.74) | (5.29) | (6.77) | (5.56) |
|  | | In favour * Left-Right |  |  |  |  |  |  | -1.87** | -2.01*** |
|  |  |  |  |  |  |  |  |  | (0.77) | (0.63) |
|  | | No information * Left-Right |  |  |  |  |  |  | -3.44*** | -2.54** |
|  |  |  |  |  |  |  |  |  | (1.31) | (1.08) |
| Business Groups ^b^ = | | |  |  |  |  |  |  |  |  |
|  | In favour | |  |  | 3.42* | 3.32** | 3.96* | 3.93** | -5.24 | -4.69 |
|  |  | |  |  | (2.06) | (1.61) | (2.06) | (1.62) | (4.29) | (3.51) |
|  | No information | |  |  | 0.99 | 0.44 | 1.08 | 0.59 | -1.47 | -0.51 |
|  |  | |  |  | (2.78) | (2.17) | (2.78) | (2.18) | (5.59) | (4.58) |
|  | In favour * Business Group Member | |  |  | 2.72 | -2.08 | 1.17 | -3.72 | -2.88 | -7.10 |
|  |  |  |  |  | (6.37) | (5.01) | (6.46) | (5.09) | (6.16) | (5.08) |
|  | No information * Business Group Member | |  |  | 9.97 | 4.46 | 11.02 | 5.32 | 13.26* | 6.34 |
|  |  |  |  |  | (7.98) | (6.23) | (8.09) | (6.33) | (7.83) | (6.42) |
|  | In favour * Left-Right | |  |  |  |  |  |  | 2.00** | 1.83*** |
|  |  |  |  |  |  |  |  |  | (0.79) | (0.65) |
|  | No information * Left-Right | |  |  |  |  |  |  | 0.50 | 0.25 |
|  |  |  |  |  |  |  |  |  | (1.05) | (0.86) |
| Sample ^c^ = | | |  |  |  |  |  |  |  |  |
|  | Danish National | | 7.85 | 2.43 | 8.68 | 3.74 | 8.17 | 2.97 | 8.01 | 5.93 |
|  |  | | (8.37) | (6.55) | (8.43) | (6.59) | (8.39) | (6.57) | (8.49) | (6.95) |
|  | Danish Regional | | 11.62* | 5.13 | 10.51* | 5.71 | 13.38** | 7.18 | 7.79 | 5.96 |
|  |  | | (6.37) | (4.98) | (6.11) | (4.78) | (6.64) | (5.20) | (6.40) | (5.24) |
|  | Dutch Municipal | | -1.97 | -5.04*** | -0.83 | -4.27** | -1.21 | -4.50*** | -2.36 | -4.46*** |
|  |  | | (2.17) | (1.70) | (2.20) | (1.73) | (2.20) | (1.73) | (2.09) | (1.72) |
|  | Dutch National | | 17.24 | 7.88 | 17.97 | 9.82 | 18.51* | 9.74 | 14.69 | 8.34 |
|  |  | | (10.92) | (8.54) | (11.25) | (8.80) | (11.17) | (8.74) | (10.52) | (8.63) |
|  | Dutch Regional | | -8.03** | -2.94 | -7.64** | -2.14 | -8.33** | -2.67 | -8.36** | -2.85 |
|  |  | | (3.77) | (2.95) | (3.84) | (3.01) | (3.83) | (3.00) | (3.61) | (2.97) |
| R-squared | | | 0.03 | 0.41 | 0.01 | 0.40 | 0.04 | 0.41 | 0.15 | 0.44 |
| N | | | 1208 | 1200 | 1197 | 1190 | 1175 | 1168 | 1161 | 1154 |

^a^ reference group: ‘Against’, ^b^ reference group: ‘Against’, ^c^ reference group ‘Danish municipal’. 0.1 > * > 0.05 > ** > 0.01 > ***

| *Table A23: Analyses including engagement in business and environmental groups 1* | | | | | | | | | | | |
| --- | --- | --- | --- | --- | --- | --- | --- | --- | --- | --- | --- |
| **Model** | | | | **A129** | **A130** | **A131** | **A132** | **A133** | **A134** | **A135** | **A136** |
| **Data** | | | | **All** | **All** | **All** | **All** | **All** | **All** | **All** | **All** |
| Intercept | | | | 48.73*** | 6.77*** | 55.40*** | 7.96*** | 50.50*** | 6.67*** | 65.65*** | 11.10*** |
|  | | | | (2.11) | (2.08) | (2.03) | (2.13) | (2.48) | (2.38) | (3.90) | (3.66) |
| Prior agreement | | | |  | 14.38*** |  | 14.58*** |  | 14.37*** |  | 13.71*** |
|  | | | |  | (0.43) |  | (0.43) |  | (0.44) |  | (0.45) |
| Environmental Group Linkage | | | | 8.14*** | 1.35 |  |  | 8.97*** | 1.41 | 5.17** | 1.18 |
|  |  |  |  | (2.18) | (1.73) |  |  | (2.22) | (1.77) | (2.27) | (1.86) |
| Business Group Linkage | | | |  |  | -4.38* | -0.26 | -6.25*** | -1.05 | -2.59 | 0.17 |
|  |  |  |  |  |  | (2.24) | (1.77) | (2.24) | (1.78) | (2.22) | (1.81) |
| Left-Right | | | |  |  |  |  |  |  | -2.88*** | -0.53 |
|  | | | |  |  |  |  |  |  | (0.59) | (0.49) |
| Environmental Groups^a^ = | | | |  |  |  |  |  |  |  |  |
|  | In favour | | | -1.33 | -0.05 |  |  | -1.22 | 0.16 | 11.12** | 11.08*** |
|  |  | | | (2.35) | (1.86) |  |  | (2.37) | (1.88) | (4.50) | (3.67) |
|  | No information | | | 2.80 | 0.08 |  |  | 3.27 | 0.40 | 9.69 | 4.48 |
|  |  | | | (3.30) | (2.61) |  |  | (3.37) | (2.67) | (6.81) | (5.55) |
|  | In favour * Environmental Group Linkage | | | 6.02* | 6.12** |  |  | 6.08* | 6.03** | 2.50 | 3.21 |
|  |  |  |  | (3.09) | (2.44) |  |  | (3.13) | (2.48) | (3.17) | (2.59) |
|  | No information * Environmental Group Linkage | | | -0.39 | 2.64 |  |  | -1.34 | 2.14 | -3.61 | 0.80 |
|  |  |  |  | (4.33) | (3.42) |  |  | (4.40) | (3.49) | (4.54) | (3.71) |
|  | In favour * Left-Right | | |  |  |  |  |  |  | -2.02*** | -1.88*** |
|  |  |  |  |  |  |  |  |  |  | (0.67) | (0.55) |
|  | No information * Left-Right | | |  |  |  |  |  |  | -1.09 | -0.70 |
|  |  |  |  |  |  |  |  |  |  | (1.03) | (0.84) |
| Business Groups^b^ = | | | |  |  |  |  |  |  |  |  |
|  | | In favour | |  |  | -0.07 | 0.84 | -0.13 | 0.77 | -1.93 | -0.63 |
|  | |  | |  |  | (1.98) | (1.55) | (1.96) | (1.55) | (3.62) | (2.95) |
|  | | No information | |  |  | -1.10 | -1.00 | -1.55 | -1.03 | -4.41 | -2.43 |
|  | |  | |  |  | (2.87) | (2.26) | (2.83) | (2.25) | (5.07) | (4.12) |
|  | | In favour * Business Group Linkage | |  |  | 3.25 | 0.85 | 3.69 | 1.24 | 4.33 | 1.50 |
|  |  |  |  |  |  | (3.18) | (2.50) | (3.18) | (2.52) | (3.13) | (2.56) |
|  | | No information * Business Group Linkage | |  |  | 1.25 | -0.49 | 2.97 | 0.75 | 3.52 | 0.73 |
|  |  |  |  |  |  | (4.49) | (3.53) | (4.48) | (3.55) | (4.47) | (3.65) |
|  | | In favour * Left-Right | |  |  |  |  |  |  | 0.35 | 0.26 |
|  | |  |  |  |  |  |  |  |  | (0.66) | (0.54) |
|  | | No information * Left-Right | |  |  |  |  |  |  | 0.59 | 0.32 |
|  |  |  |  |  |  |  |  |  |  | (0.94) | (0.76) |
| Sample^c^ = | | | |  |  |  |  |  |  |  |  |
|  | | | Danish National | 7.35 | 5.72 | 9.22 | 4.66 | 7.84 | 5.93 | 7.94 | 8.16 |
|  | | |  | (7.31) | (5.75) | (7.23) | (5.66) | (7.32) | (5.77) | (7.70) | (6.26) |
|  | | | Danish Regional | 10.07** | 3.01 | 6.13 | 1.53 | 9.04* | 3.23 | 3.15 | 1.36 |
|  | | |  | (4.83) | (3.85) | (4.75) | (3.76) | (5.07) | (4.05) | (5.03) | (4.14) |
|  | | | Dutch Municipal | -2.40 | -5.05*** | -1.66 | -4.59*** | -2.81 | -4.99*** | -2.86* | -4.83*** |
|  | | |  | (1.75) | (1.39) | (1.79) | (1.41) | (1.78) | (1.42) | (1.74) | (1.42) |
|  | | | Dutch National | 12.77 | 1.86 | 11.52 | 3.23 | 12.52 | 2.94 | 7.32 | 1.03 |
|  | | |  | (9.73) | (7.65) | (10.10) | (7.91) | (9.97) | (7.87) | (9.67) | (7.86) |
|  | | | Dutch Regional | -7.29** | -2.09 | -6.27* | -1.25 | -8.21*** | -2.16 | -7.80** | -2.20 |
|  | | |  | (3.14) | (2.47) | (3.20) | (2.52) | (3.17) | (2.51) | (3.07) | (2.50) |
| R-squared | | | | 0.04 | 0.41 | 0.01 | 0.39 | 0.04 | 0.41 | 0.11 | 0.42 |
| N | | | | 1860 | 1843 | 1845 | 1830 | 1812 | 1797 | 1794 | 1779 |

^a^ reference group: ‘Against’, ^b^ reference group: ‘Against’, ^c^ reference group ‘Danish municipal’. 0.1 > * > 0.05 > ** > 0.01 > ***

| *Table A24: Analyses including engagement in business and environmental groups 2* | | | | | | | | | | |
| --- | --- | --- | --- | --- | --- | --- | --- | --- | --- | --- |
| **Model** | | | **A137** | **A138** | **A139** | **A140** | **A141** | **A142** | **A143** | **A144** |
| **Data** | | | **MC** | **MC** | **MC** | **MC** | **MC** | **MC** | **MC** | **MC** |
| Intercept | | | 50.18*** | 7.58*** | 55.52*** | 7.91*** | 50.15*** | 6.36** | 69.26*** | 13.92*** |
|  | | | (2.57) | (2.56) | (2.48) | (2.62) | (3.01) | (2.93) | (4.49) | (4.38) |
| Prior agreement | | |  | 14.23*** |  | 14.54*** |  | 14.09*** |  | 13.25*** |
|  | | |  | (0.52) |  | (0.53) |  | (0.54) |  | (0.56) |
| Environmental Group Linkage | | | 7.19*** | 1.99 |  |  | 8.49*** | 2.68 | 5.82** | 3.27 |
|  |  |  | (2.63) | (2.08) |  |  | (2.66) | (2.12) | (2.66) | (2.19) |
| Business Group Linkage | | |  |  | -5.90** | -0.62 | -7.87*** | -2.06 | -3.19 | -0.24 |
|  |  |  |  |  | (2.76) | (2.18) | (2.75) | (2.19) | (2.69) | (2.22) |
| Left-Right | | |  |  |  |  |  |  | -3.84*** | -1.22** |
|  | | |  |  |  |  |  |  | (0.68) | (0.57) |
| Environmental Groups ^a^ = | | |  |  |  |  |  |  |  |  |
|  | | In favour | -3.28 | -0.82 |  |  | -2.65 | -0.31 | 8.12 | 10.89** |
|  | |  | (2.86) | (2.26) |  |  | (2.86) | (2.27) | (5.25) | (4.31) |
|  | | No information | -4.37 | -2.43 |  |  | -3.34 | -1.44 | 11.90 | 10.13 |
|  | |  | (4.36) | (3.45) |  |  | (4.51) | (3.60) | (9.01) | (7.41) |
|  | | In favour * Environmental Group Linkage | 9.77*** | 8.15*** |  |  | 9.88*** | 8.04*** | 4.89 | 4.33 |
|  |  |  | (3.76) | (2.97) |  |  | (3.78) | (3.00) | (3.79) | (3.11) |
|  | | No information * Environmental Group Linkage | 10.20* | 7.35* |  |  | 8.44 | 6.07 | 0.43 | 1.08 |
|  |  |  | (5.64) | (4.46) |  |  | (5.78) | (4.61) | (6.08) | (5.02) |
|  | | In favour * Left-Right |  |  |  |  |  |  | -1.57** | -1.81*** |
|  |  |  |  |  |  |  |  |  | (0.78) | (0.64) |
|  | | No information * Left-Right |  |  |  |  |  |  | -2.42* | -1.90* |
|  |  |  |  |  |  |  |  |  | (1.35) | (1.12) |
| Business Groups ^b^ = | | |  |  |  |  |  |  |  |  |
|  | In favour | |  |  | 1.92 | 2.79 | 2.53 | 3.11 | -6.53 | -5.37 |
|  |  | |  |  | (2.43) | (1.91) | (2.39) | (1.90) | (4.33) | (3.55) |
|  | No information | |  |  | 0.85 | -0.01 | 0.51 | -0.04 | -3.58 | -1.70 |
|  |  | |  |  | (3.28) | (2.57) | (3.22) | (2.55) | (5.59) | (4.58) |
|  | In favour * Business Group Linkage | |  |  | 4.46 | 0.58 | 5.34 | 1.66 | 4.18 | 0.50 |
|  |  |  |  |  | (4.04) | (3.18) | (4.00) | (3.18) | (3.92) | (3.23) |
|  | No information * Business Group Linkage | |  |  | 3.51 | 2.72 | 5.65 | 4.22 | 7.45 | 5.01 |
|  |  |  |  |  | (5.37) | (4.21) | (5.31) | (4.21) | (5.34) | (4.38) |
|  | In favour * Left-Right | |  |  |  |  |  |  | 1.96** | 1.81*** |
|  |  |  |  |  |  |  |  |  | (0.80) | (0.66) |
|  | No information * Left-Right | |  |  |  |  |  |  | 0.72 | 0.31 |
|  |  |  |  |  |  |  |  |  | (1.07) | (0.88) |
| Sample ^c^ = | | |  |  |  |  |  |  |  |  |
|  | Danish National | | 5.63 | 1.29 | 8.35 | 3.08 | 5.58 | 1.26 | 4.79 | 3.68 |
|  |  | | (8.26) | (6.49) | (8.40) | (6.59) | (8.22) | (6.51) | (8.46) | (6.94) |
|  | Danish Regional | | 13.68** | 6.40 | 9.65 | 5.36 | 14.56** | 8.30 | 9.13 | 7.20 |
|  |  | | (6.30) | (4.96) | (6.10) | (4.78) | (6.55) | (5.19) | (6.43) | (5.27) |
|  | Dutch Municipal | | -2.22 | -5.18*** | -1.61 | -4.57*** | -2.45 | -5.00*** | -2.95 | -4.82*** |
|  |  | | (2.14) | (1.69) | (2.21) | (1.74) | (2.18) | (1.73) | (2.11) | (1.74) |
|  | Dutch National | | 19.11* | 8.89 | 15.38 | 9.33 | 17.90 | 10.19 | 15.45 | 9.45 |
|  |  | | (10.81) | (8.50) | (11.34) | (8.89) | (11.10) | (8.79) | (10.61) | (8.71) |
|  | Dutch Regional | | -8.51** | -3.25 | -8.41** | -2.56 | -9.83*** | -3.55 | -9.27** | -3.49 |
|  |  | | (3.72) | (2.94) | (3.84) | (3.02) | (3.76) | (2.99) | (3.60) | (2.97) |
| R-squared | | | 0.06 | 0.42 | 0.02 | 0.40 | 0.07 | 0.42 | 0.16 | 0.44 |
| N | | | 1208 | 1200 | 1197 | 1190 | 1175 | 1168 | 1161 | 1154 |

^a^ reference group: ‘Against’, ^b^ reference group: ‘Against’, ^c^ reference group ‘Danish municipal’. 0.1 > * > 0.05 > ** > 0.01 > ***

**References**

Montanaro, L. (2012). The Democratic Legitimacy of Self-Appointed Representatives. *Journal of Politics* *74*(4), 1094-1107.

Saward, M. (2008). Representation and Democracy: Revisions and Possibilities. *Sociology Compass* *2*(3), 1000-1013.

Simon, Herbert. A. (1991). Bounded Rationality and Organizational Learning. *Organization Science*, *2*(1), 125-134.

Tajfel, H., & Turner, J.C. (1986). The social identity theory of intergroup behaviour. In S. Worchel & W. G. Austin (Eds.), *Psychology of Intergroup Relations* (pp. 7–24). Nelson-Hall.
